# Computational Screening of Chalcogen-Terminated Inherent Multilayer MXenes and M_2_AX Precursors

**DOI:** 10.1021/acs.inorgchem.4c01690

**Published:** 2024-08-26

**Authors:** Pernilla Helmer, Jonas Björk, Johanna Rosen

**Affiliations:** Department of Physics, Chemistry and Biology, Linköping University, Olaus Magnus väg 37, 583 30 Linköping, Sweden

## Abstract

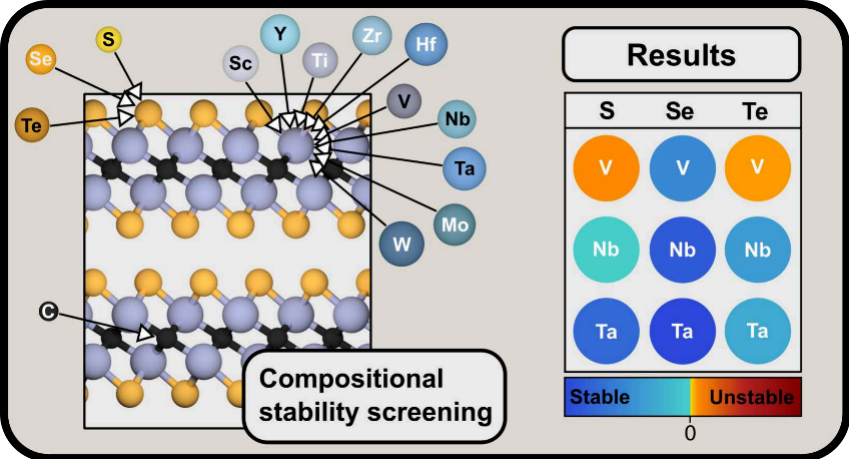

Sulfur-terminated single sheet (ss-)MXene was recently achieved by delamination of multilayered van der Waals bonded (vdW)-MXenes Nb_2_CS_2_ and Ta_2_CS_2_ synthesized through solid-state synthesis, rather than via the traditional way of selectively etching A-layers from the corresponding MAX phase. Inspired by this, we perform a computational screening study of vdW-MXenes M_2_CCh_2_ isotypical to Nb_2_CS_2_ and Ta_2_CS_2_, with M = Sc, Y, Ti, Zr, Hf, V, Nb, Ta, Mo, or W and Ch = S, Se, or Te. The thermodynamic stability of each vdW-MXene M_2_CCh_2_ is assessed, and the dynamical stability of both vdW- and ss-MXene is considered through phonon dispersions. We predict seven stable vdW-MXenes, out of which four have not been reported previously, and one, V_2_CSe_2_, incorporates a new transition metal element into this family of materials. Electronic properties are presented for the vdW- and ss-forms of the stable vdW-MXenes, suggesting that the materials are either metallic, semimetallic, or semiconducting. In previous experimental reports the vdW-MXene Nb_2_CS_2_ is synthesized by manipulation of the corresponding M_2_AX phase Nb_2_SC. Therefore, we also evaluate the thermodynamic stability of the corresponding M_2_AX phases, identifying 15 potentially stable phases. Six of these are experimentally reported, leaving nine new M_2_AX phases for future experimental investigation.

## Abstract 2

Following the experimental delamination of two van der Waals (vdW) bonded multilayered MXenes with chalcogen terminations into single sheet (ss-)MXene, we perform a computational screening study to identify further stable vdW-MXenes. We predict seven vdW-MXenes, out of which four have not been experimentally reported to date. We assess the potential for delamination into 2D by evaluating the interlayer binding energies, suggesting that all predicted vdW-MXenes are likely possible to be delaminated into 2D.

## Introduction

Simply because of their lowered dimensionality, 2D materials often display properties that differ significantly from those of their 3D counterparts. This makes them an intriguing group of materials, and they are as a consequence gaining increasingly more interest from the research community. Furthermore, their large surface-to-volume or surface-to-weight ratio makes them interesting for any application where the material’s surface is a significant factor, e.g., for charge storage or catalysis.^1,2^

**Figure 1:**
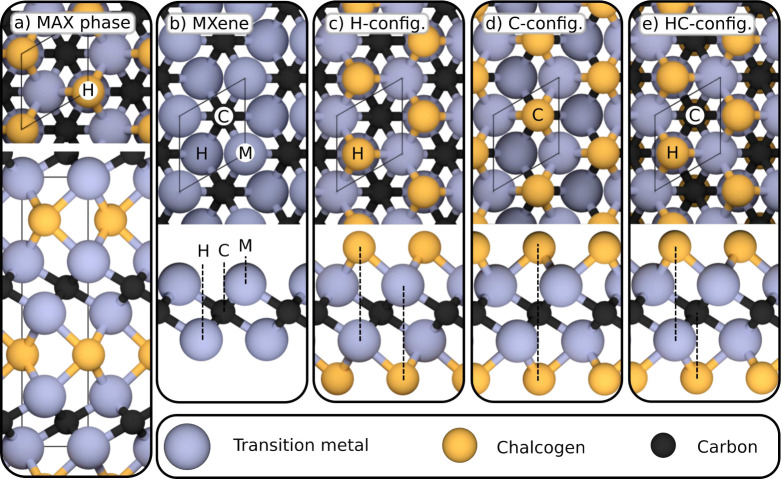
Schematic MAX and MXene structures. (a) M_2_AX structure, here with M being a transition metal, A a chalcogen, and X carbon. (b) Unterminated MXene, with possible termination sites indicated: hollow site (H), carbon site (C), and metal site (M). (c) MXene terminated on the H-site (H-configuration). (d) MXene terminated on the C-site (C-configuration). (e) MXene terminated on the C- and H-site in the top and bottom layer, respectively (HC-configuration).

One family of 2D materials that is currently receiving intense interest from the materials research community is the family of the so-called MXenes. A MXene consists of an odd number of alternating M and X layers on the form M_*n*+1_X_*n*_, where M stands for a transition metal and X stands for carbon and/or nitrogen, and is traditionally synthesized by selective etching of layers of so-called A elements (e.g., Al, Si, or Ge) from an atomically layered parent MAX phase of the general formula M_*n*+1_AX_*n*_.^3^ The MAX structure for *n* = 1 is shown schematically in Figure 1a with X = C, and A as a chalcogen shown in yellow. The structure for the corresponding MXene is shown in Figure 1b. Upon etching of the A layers, so-called surface terminations will attach to the exposed transition metal surfaces,^4^ resulting in a multilayered structure (ml-MXene) in which the individual terminated MXene sheets are bound together by weak interlayer interactions, as opposed to the relatively strong bonding between the MXene units and A-element in a MAX phase. The individual sheets of the ml-MXene can be successively delaminated into single sheet (ss-)MXene,^4^ similarly to how other 2D materials are realized by delamination of the respective parent phase with weak interlayer bonding.^5^ Thus, the synthesis of MXene typically involves an additional step compared to the delamination of van der Waals compounds.

The surface terminations influence the properties of the resulting MXene^4,6^ and are thus highly important when considering MXenes for applications. In principle, the MXene properties can be tuned by tailoring the surface terminations. However, in practice, this is far from trivial, since typical termination species—O, F, and hydroxyl groups—are all present during the most common methods of etching and are not necessarily interchangeable post etching.^6^ Nevertheless, recent advancements have been made for controlling surface terminations. For instance, it has been shown that MAX phases etched in Br-based molten salts result in Br-terminated MXenes, where Br in turn can be substituted for a number of other terminations post etching.^6^

Besides ml-MXene synthesized by selective etching of the corresponding MAX phase, the ml-MXenes Ta_2_CS_2_ and Nb_2_CSe_2_ have been reported as synthesized directly by solid state synthesis in 1970 and 2020,^7,8^ respectively, and ml-MXene Nb_2_CS_2_ has been synthesized by manipulation of the corresponding MAX phase, for the first time reported in 1992.^9^ Ta_2_CSe_2_ has also been mentioned in the literature,^10^ although, to the best of our knowledge, no experimental evidence of its existence has been published. We will therefore consider it as not reported to date.

It is worth noting that the MXene concept was not coined until 2011, and before that the chalcogen-terminated vdW-MXenes were termed van der Waals type carbosulfides or sulfide carbides^10,11^ and at the time of first report simply as complex carbides (“Komplexcarbid”).^7^ They have also recently been termed transition metal carbochalcogenices (TMCCs), argued to be a combination of MXenes and another intriguing family of 2D materials known as transition metal dichalcogenides (TMDs).^8,12^ In this work, we will refer to them as chalcogen-terminated van der Waals bonded multilayer MXenes or simply vdW-MXenes. The vdW-MXenes are synthesized by a completely different process compared to traditional ml-MXene and are true van der Waals (vdW) bonded solids, as well established in the literature,^13^ without even the possibility for trace defects coming from the etching process required in traditional ml-MXene synthesis. Therefore, we choose to call them vdW-MXene to highlight their fundamental differences from traditional ml-MXene synthesized via selective etching. Although the first of these chalcogen-terminated vdW-MXenes was reported in 1970, it was only recently that any of them were delaminated into single sheet (ss-)MXene.^12^ More specifically, Nb_2_CS_2_ and Ta_2_CS_2_ were delaminated into ss-MXene, thus realizing a novel method to synthesize high quality sulfur-terminated ss-MXenes, excluding the need for both selective etching and termination substitution. Given the absence of multiple elemental species in the reaction path of ss-MXene synthesis from vdW-MXenes comparend to from selective etching of a corresponding MAX phase, it is reasonable to expect fewer defects in ss-MXene synthesized from vdW-MXenes.

Chalcogen-terminated MXenes in both vdW bonded bulk and single sheet forms have been studied previously from both theoretical and experimental perspectives, considering, e.g., superconductivity,^6^ gas sensing,^14^ catalysis,^8^ and for application as anode material in different ion batteries.^15,16^ High quality chalcogen-terminated ss-MXenes may also be interesting for use in van der Waals heterostructures. These composite architectures, composed of different single sheet 2D materials stacked together, have been shown to display a plethora of interesting properties, with potential for manipulation of electronic properties, surface reconstruction, tunneling devices, and various light-interacting devices.^17^ Thus, vdW-MXenes isostructural to vdW-Ta_2_CS_2_ and vdW-Nb_2_CS_2_ are intriguing, and an in-depth investigation of these systems is indeed motivated.

Inspired by the recent report on delamination of vdW-Nb_2_CS_2_ and vdW-Ta_2_CS_2_ into their respective single sheet counterparts,^12^ we have performed a systematic analysis of the formation enthalpy for the phases isostructural to vdW-Ta_2_CS_2_, vdW-Nb_2_CS_2_, and vdW-Nb_2_CSe_2_ on the form M_2_CCh_2_, where M = Sc, Y, Ti, Hf, Zr, V, Nb, Ta, Mo, or W is a transition metal, and Ch = S, Se, or Te is a chalcogen. Further, we have asserted the dynamical stability of the vdW-MXenes found to have a negative formation enthalpy, thereby predicted as thermodynamically stable, by evaluation of the respective phonon dispersions for both the vdW- and ss-MXene of each chemical system. We have also calculated the corresponding electronic properties, finding the MXenes to be either metallic, semimetallic, or semiconducting depending on termination configuration and dimensionality. Nb_2_CS_2_, one of the so far delaminated vdW-MXenes, is synthesized by successive manipulation of the corresponding MAX phase by formation of the intermediate structure Cu_*x*_Nb_2_CS_2_, followed by removal of Cu^9,11,12^ Because of this, we also assess all M_2_AX phases on the form M_2_ChC within the studied compositional space. This is done both in an attempt to identify differences between the Ta–C–S, Nb–C–Se, and Nb–C–S chemical systems and to assert the identification of any system reminiscent of Nb–C–S, for which the vdW-M_2_CCh_2_ phase may only be indirectly synthesizable via the corresponding M_2_AX phase.

## Results and Discussion

### vdW-MXene Structure

Phases in the considered chemical systems were extracted from the Materials Project database and successively relaxed,^18,19^ taking van der Waals interactions into account. Within this set of phases, the experimentally reported vdW-MXenes Nb_2_CS_2_ and Ta_2_CS_2_ were included and used to construct an initial prototype structure for the vdW-MXene phase of the remaining elemental systems considered. To optimize the vdW-MXene structure for each system, the most likely termination sites were identified by mapping the energy landscape of the four selected phases Nb_2_CS_2_, Nb_2_CTe_2_, Ta_2_CS_2_, and V_2_CSe_2_, chosen among those with lowest formation enthalpy considering the initial prototype vdW-MXene structures and including two of the experimentally reported phases.

To map the energy for different termination sites, the ss-MXene unit cell, indicated in Figure 1b, was divided into 6 by 6 positions for Ch termination, resulting in 36 termination configurations for each phase, out of which several were equivalent by symmetry. The structures were then fully relaxed, and the final position of the Ch-termination was determined by the (shortest) C–Ch distance. In this way, three possible termination sites were identified for the four probed structures, indicated in Figure 1b: carbon site (C), hollow site (H), and metal site (M). The resulting energy maps are shown in Figure S1.

Without exception, configurations with termination at the M-site, termed the M-configuration in the following, were only stable during relaxation under imposed symmetry constraints. When the symmetry constraints were released, the M-configuration relaxed into termination at the H-site (H-configuration) or the C-site (C-configuration). This has been shown previously for a range of terminations, including sulfur.^20^ Hence, the M-configuration was not considered further. The preference for the H- or C-site may be understood through the difference in electronegativity of the different elements, as discussed in the Supporting Information.

The H- and C-configurations, with terminations at the H- and C-sites, respectively, are depicted in Figures 1c and 1d. In addition to full population at either the H- or C-site, population of the H-site on one side and C-site on the other side of the MXene sheet was also considered, inspired by previous work.^16,21−23^ This alternating configuration is in the following termed the HC-configuration, to indicate the alternating population of the H- and C-sites. The HC-configuration is shown in Figure 1e.

Several different stackings of the differently terminated ss-MXene units were considered to model the vdW-MXene, with the general result that the specific stacking is overall less important for the total energy than the termination configuration is although it is of the same order for many compositions. This agrees with intuition, given that the vdW-MXenes are expected to have weak interlayer interactions. Thus, we leave the detailed discussion on stacking methodology for the interested reader to find in the Supporting Information.

### Screening of Formation Enthalpy

**Figure 2:**
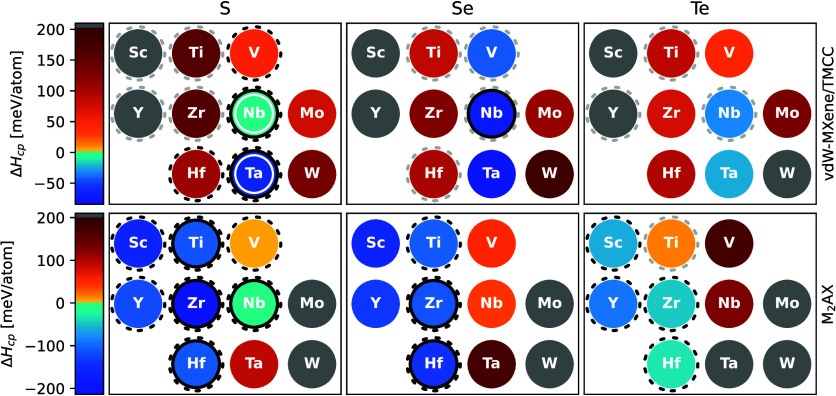
Formation enthalpy heatmap. Different colors show formation enthalpy with respect to competing phases Δ*H*_cp_ for vdW-MXenes M_2_CCh_2_ and corresponding M_2_AX phases M_2_ChC for the indicated transition metals M and chalcogens Ch. Blue colors show negative formation enthalpies, indicating thermodynamic stability with respect to competing phases, while red colors show positive formation enthalpies, indicating thermodynamic instability or metastability. Phases shown in yellow are only just above zero formation enthalpy. Phases shown in gray are considered far from being stable, with Δ*H*_cp_ > 200 meV/atom. Dashed and solid black circles indicate phases theoretically predicted stable in previous work^22,24^ and experimentally reported,^7−9,12,25−28^ respectively. Gray dashed circles indicate theoretical work without rigorous stability analysis or that a phase has been predicted as close to stable.^14−16,21,23,29−32^ White solid circles indicate that the vdW-MXene has been delaminated into ss-MXene.^12^

The results from the computational screening study are summarized in Figure 2, where the formation enthalpy with respect to competing phases Δ*H*_cp_, as defined in eq 1 of the Computational Methods section, is displayed as a heatmap. The upper row shows the vdW-MXene with the lowest formation enthalpy for the respective composition, and the lower row shows the formation enthalpy for the corresponding M_2_AX phases. Blue colors indicate negative formation enthalpies, implying thermodynamic stability, while orange and red colors imply positive formation enthalpies and thus thermodynamic instability or metastability. Phases shown in yellow have a small positive formation enthalpy (Δ*H*_cp_ < 30 meV/atom)^33^ and could possibly be synthesized, although here predicted just unstable. Gray color indicates that a phase is far from stability, defined as Δ*H*_*cp*_ > 200 meV/atom.

Fifteen of the MAX phases have a negative formation enthalpy, Δ*H*_cp_ < 0, and are thus predicted to be thermodynamically stable. In addition, we also identify two phases, V_2_SC and Ti_2_TeC, as having small positive formation energies (Δ*H*_cp_ = 8 and 16 meV/atom, respectively) and thus being close to thermodynamically stable. More intriguingly, we find seven vdW-MXenes with Δ*H*_*c*p_ < 0, indicating that these phases are possible to synthesize directly by solid synthesis, analogous to the synthesis of Ta_2_CS_2_ and Nb_2_CSe_2_. Out of these, four have to the best of our knowledge neither been previously reported experimentally nor theoretically predicted following a rigorous stability analysis. By rigorous stability analysis, we mean the assessment of dynamical stability and thermodynamical stability with respect to competing phases. More specifically, these are Ta_2_CSe_2_, Nb_2_CTe_2_, Ta_2_CTe_2_, and V_2_CSe_2_, i.e., three more phases with the same transition metals previously reported for the chalcogen-terminated vdW-MXenes and one phase, V_2_CSe_2_, incorporating a new transition metal from the same group.

We can see that the vdW-MXenes predicted to be stable all have a transition metal element from group V of the periodic table. The MAX phases are a little less restrictive when it comes to stable compositions and include transition metal elements primarily from the periodic table groups III and VI, and one from group V. Further, those that are predicted unstable and include a transition metal from group V are closer to being stable, i.e., have a Δ*H*_cp_ closer to zero, compared to those with a transition metal from group VI. There is also a trend of higher stability of the MAX phases for smaller chalcogen size, with the number of phases predicted stable being 6, 5, and 4 for the set of MAX phases including Ch = S, Se, and Te, respectively. This can be correlated to the degree of packing in the structures, which is further discussed in relation to Figure S3. A similar trend is not evident for the vdW-MXenes.

As mentioned previously, the experimentally reported phase Nb_2_CS_2_ has to date not been reported as synthesized directly by solid state synthesis, but only by manipulation of the corresponding M_2_AX phase. This is something that has been interpreted as Nb_2_CS_2_ being metastable in earlier reports.^9^ In Figure 2, on the other hand, both the vdW-MXene and MAX phases of the Nb–C–S system are predicted to have a negative formation enthalpy. However, this should not be interpreted as a discrepancy between experimental work and calculations since Nb_2_CS_2_ is predicted to be stable by less than 1 meV/atom, which is well within the expected error for these calculations. The results do indicate, however, that both the MAX phase and the vdW-MXene of the Nb–C–S system have a close-to-zero formation enthalpy, which is not observed in any of the other chemical systems considered. This suggests a low probability that any of the other considered vdW-MXene phases can be synthesized from the corresponding MAX phase similarly to how Nb_2_CS_2_ is synthesized from Nb_2_SC, although a thorough study of the chemical processes during synthesis of Nb_2_CS_2_ is needed in order to decisively identify the criteria for this synthesis route. Nevertheless, it should be noted that MAX phases predicted to have formation enthalpies well above zero have been synthesized by elemental substitution in the A-layer.^33,34^ With this in mind, chemical systems suitable for further investigation could be Ti–C–Se, Zr–C–Te, Hf–C–Se, and Hf–C–Te. In these systems, the M_2_AX phase is stable, and Δ*H*_cp_ < 100 meV/atom for the vdW-MXene, being 85, 72, 98, and 95 meV/atom, respectively. The V–C–S and Ti–C–Te systems could also be of interest, in which the M_2_AX phase is predicted as close to stable with Δ*H*_cp_ = 8 and 16 meV/atom, respectively, and the vdW-MXene phase with Δ*H*_cp_ = 48 and 88 meV/atom. In all other systems where the M_2_AX phase is predicted to be stable, Δ*H*_cp_ > 100 meV/atom for the vdW-MXene.

**Figure 3:**
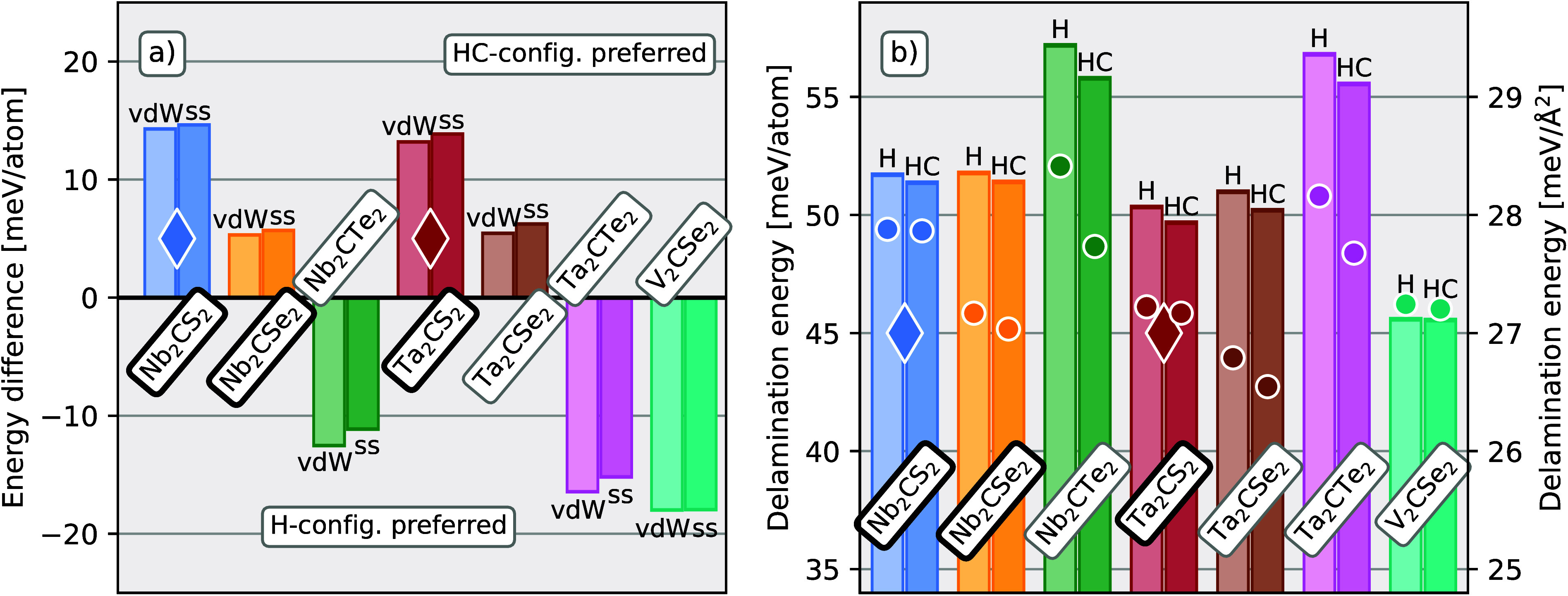
Energy comparisons of the different termination configurations and dimensionality. (a) Energy difference between the H- and HC-configurations for the seven vdW-MXenes identified as having Δ*H*_cp_ < 0 and their corresponding ss-MXenes. Positive values indicate preference for the HC-configuration and negative for the H-configuration. The brighter, leftmost bar for each pair of color bars indicates the vdW-MXene phase for the most beneficial stacking, while the darker, rightmost bar indicates the delaminated ss-MXene. (b) Delamination energy for the H- and HC-configurations for the seven vdW-MXene phases with Δ*H*_cp_ < 0 as measured in meV/atom (bars) and meV/Å^2^ (circles). Experimentally reported phases are indicated by a black border around the chemical formula, and phases are delaminated into ss-MXene by a diamond shape.

Out of the vdW-MXenes predicted as thermodynamically stable, all were found to prefer either the H- or HC-configuration. Thus, the following discussion will be focused on these two configurations. The difference between the two configurations ranges from ∼5 to ∼20 meV/atom. The lighter colored bars of Figure 3a) show the energy difference per atom between the two configurations for the seven vdW-MXenes predicted stable. Positive values indicate preference for the HC-configuration and negative for the H-configuration. The three phases previously reported in the literature, i.e., Nb_2_CCh_2_ with Ch = S or Se and Ta_2_CS_2_, prefer the HC-configuration, as does Ta_2_CSe_2_. The three remaining phases with Δ*H*_cp_ < 0, i.e., Nb_2_CTe_2_, Ta_2_CTe_2_, and V_2_CSe_2_, prefer the H-configuration. The two phases reported as delaminated into ss-MXene, indicated by diamond shapes, show the strongest preference for the HC-configuration.

Since we are ultimately interested in the delaminated ss-MXene, we have also considered the ss-MXene phase in addition to the vdW-MXene bulk phase. The darker bars in Figure 3a show the energy difference per atom between the H- and HC-configurations for ss-MXenes. These energy differences are very similar between the vdW-MXene and ss-MXene phases; i.e., stacking of the ss-MXenes into bulk vdW-MXene does not significantly affect the preferred configuration of the terminations, although a slight shift toward a stronger preference for the H-configuration is observed for the ss-MXenes. Further, we can see that the preference for the HC-configuration decreases with increasing chalcogen size, regardless of dimensionality. The difference between the two configurations is very similar between any two Nb_2_CCh_2_ and Ta_2_CCh_2_ sharing the same chalcogen element, while V_2_CSe_2_ shows a clear preference for the H-configuration, in contrast to (Nb/Ta)_2_CSe_2_. No clear trend between termination configuration and transition metal elements has been identified.

In Figure 3b, the delamination energy, defined as the energy difference per atom between the ss-MXene and the vdW-MXene, is shown by the colored bars for both the H- and HC-configuration for the seven vdW-MXenes predicted stable. The delamination energy ranges from 46 meV/atom for V_2_CSe_2_ to 57 meV/atom for Nb_2_CTe_2_, differing very little between the H- and HC-configurations. For M = Nb or Ta, the delamination energy is very similar between Ch = S or Se, while for Ch = Te, a slight increase of ∼10% is observed. When instead considering the delamination energy in meV/Å^2^, shown by the circles in Figure 3b, a decrease in delamination energy is seen between the S- and Se-terminated structures caused by the larger lattice parameter of the Se-terminated structures. For comparison with other vdW laminated structures, the reader is referred to the very comprehensive screening study by Mounet et al.,^13^ where Nb_2_CS_2_ and Ta_2_CS_2_ are included, as well as a large number of well-known vdW-bonded laminated structures.

Although the trends of the delamination energies differ slightly depending on the considered unit, they are similar across all considered phases. Given that two of the phases have been delaminated experimentally, indicated by the two diamond shapes, the similar delamination energies between compositions suggest that it is likely that all are possible to delaminate if the respective vdW-MXene can be realized. In particular, the vdW-MXene incorporating a new transition metal, V_2_CSe_2_, is among the phases with lowest delamination energy, indicating that it is relatively easy to delaminate. Again, experimentally reported vdW-MXenes are indicated by a black border around the chemical formula.

In Figure 2, phases which have previously been experimentally reported are indicated by black solid circles,^7,9,12,25−28,35^ while phases which have been theoretically predicted or in some other way considered from a stability perspective are indicated by dashed black and gray circles, respectively. With regard to theoretical studies, black indicates that the phase has been predicted stable by formation enthalpy analysis and dynamical stability analysis thorough phonon dispersion,^22,24^ while gray indicates that the phase has been studied to some extent but that a rigorous stability analysis including formation enthalpy with respect to competing phases is missing^14−16,21,23,29−32^ or predicts the phase as nearly stable.^24^

Our results are in agreement with experimental results regarding the considered MAX_2_ phases, in that all experimentally reported structures are predicted as thermodynamically stable. They are also in agreement with experiments for the vdW-MXene phases, with the exception of Nb_2_CS_2_ which we predict as just stable, although experimental reports have interpreted the phase as metastable.^9^ Further, our results are to a large extent consistent with existing previous theoretical reports, while identified discrepancies for the vdW-MXenes Hf_2_CS_2_ and V_2_CS_2_, which have previously been predicted as thermodynamically stable, can be attributed to differences in the sets of competing phases used here and in previous work. Tables over the sets of most competing phases identified in this work, and a more in-depth discussion on our work in relation to previous reports, can be found in the Supporting Information.

We have also compared our results for the preferred termination configurations and stacking sequences with those from previous theoretical work, which are in complete agreement. For a detailed discussion on these topics, the reader is again referred to the Supporting Information.

### Electronic Properties and Dynamical Stability

For the seven vdW-MXenes that we predict to be thermodynamically stable, the electronic properties have been evaluated, and the dynamical stability has been established through calculation of phonon dispersions.

#### V_2_CSe_2_

We start the discussion with the novel phase V_2_CSe_2_. As mentioned, this is the first prediction of the existence of this vdW-MXene phase, and we find the H-configuration to have the lowest formation enthalpy for this composition. The energy required to delaminate the vdW-V_2_CSe_2_ into ss-MXene is computed to 46 meV/atom, as seen in Figure 3b. This is marginally lower than the two experimentally delaminated phases Nb_2_CS_2_ and Ta_2_CS_2_, both with a delamination energy calculated to ∼50 meV/atom. Thus, there is good reason to expect that V_2_CSe_2_ can also be delaminated into ss-MXene.

**Figure 4:**
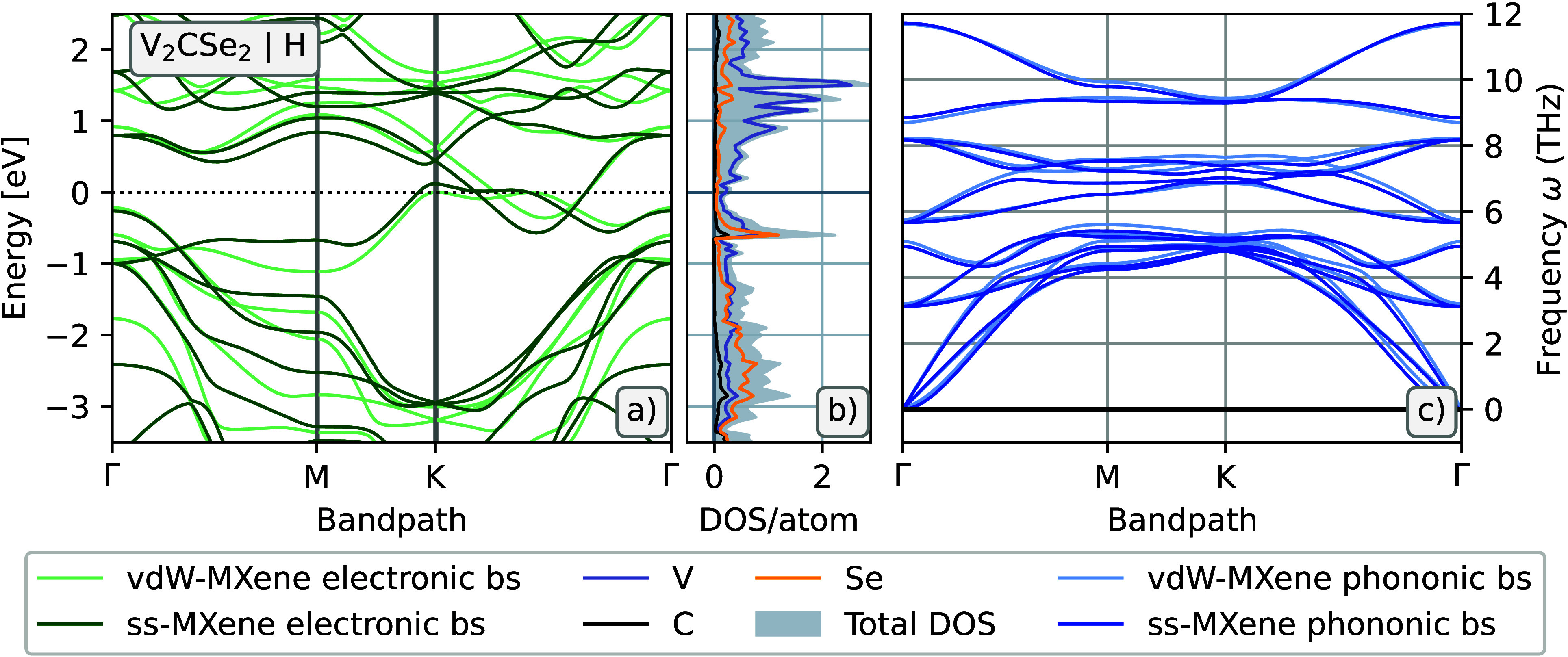
Properties for V_2_CSe_2_. (a) Electronic and (c) phononic band structures for the predicted vdW-MXene phase V_2_CSe_2_ (light colors) in the H-configuration (indicated in a) by the letter H displayed next to the chemical formula) and its delaminated ss-MXene drivative (dark colors). (b) Partial DOS for ss-V_2_CSe_2_ in the H-configuration.

The electronic band structure and partial DOS and phonon spectra for V_2_CSe_2_ are displayed in Figure 4a–c. The electronic band structure in Figure 4a, shown in dark green for ss-MXene and in lighter green for vdW-MXene, indicates a metallic material regardless of dimensionality. The partial DOS in Figure 4b refers to the ss-MXene, and it displays a large peak at 0.6 eV below the Fermi level, corresponding to strong hybridization between V and Se, and a minimum around the Fermi level. At 1–3 eV below the Fermi level there is a broader area of several peaks showing hybridization of V and Se at higher energies and also between all three elements at lower energies within this interval. At–3.8 eV below the Fermi level (not shown), there is strong hybridization of C and V. The lack of negative (imaginary) frequencies in the phonon dispersion, shown in Figure 4c, implies the dynamical stability of both vdW-V_2_CSe_2_ (light blue) and ss-V_2_CSe_2_ (dark blue).

V_2_CSe_2_ is also found to be dynamically stable in the HC-configuration, although this configuration has a higher formation enthalpy by just shy of 20 meV/atom. For completeness, the electronic band structure has been evaluated also for this configuration, which proved to be semiconducting with a very small indirect bandgap of 0.05 eV for vdW-V_2_CSe_2_ and 0.19 eV for ss-V_2_CSe_2_. Using the modified Becke–Johnssons (mBJ) functional, the bandgap closes for the vdW-MXene and shrinks to 0.12 eV for the ss-MXene. The electronic band structure and DOS and phonon band structure for the HC-terminated V_2_CSe_2_ can be seen in Figure S4.

Although this is the first report on vdW-V_2_CSe_2_, ss-V_2_CSe_2_ has been studied theoretically by two previous reports. Tang et al.^15^ investigated V_2_CCh_2_ MXene with Ch = S, Se, and Te for use as anode material in Li-ion batteries, and Wang et al.^16^ considered V_2_CSe_2_ specifically for anode material in non-Li-ion batteries. Both report the material as being interesting for use as an anode material in next-generation batteries. However, as pointed out earlier, neither of these reports consider the vdW-MXene phase but only the ss-MXene, nor do they consider the stability of the phases with respect to competing phases but merely dynamical stability. The phonon dispersion presented in Figure 4c is in good agreement with both previous reports, although there are some discrepancies in the dispersion of the optical branches between the current and previous work. The electronic properties calculated here also agree with those presented earlier, finding V_2_CSe_2_ to be metallic.^15,16^

#### M_2_CCh_2_ with M = Nb, Ta

**Figure 5:**
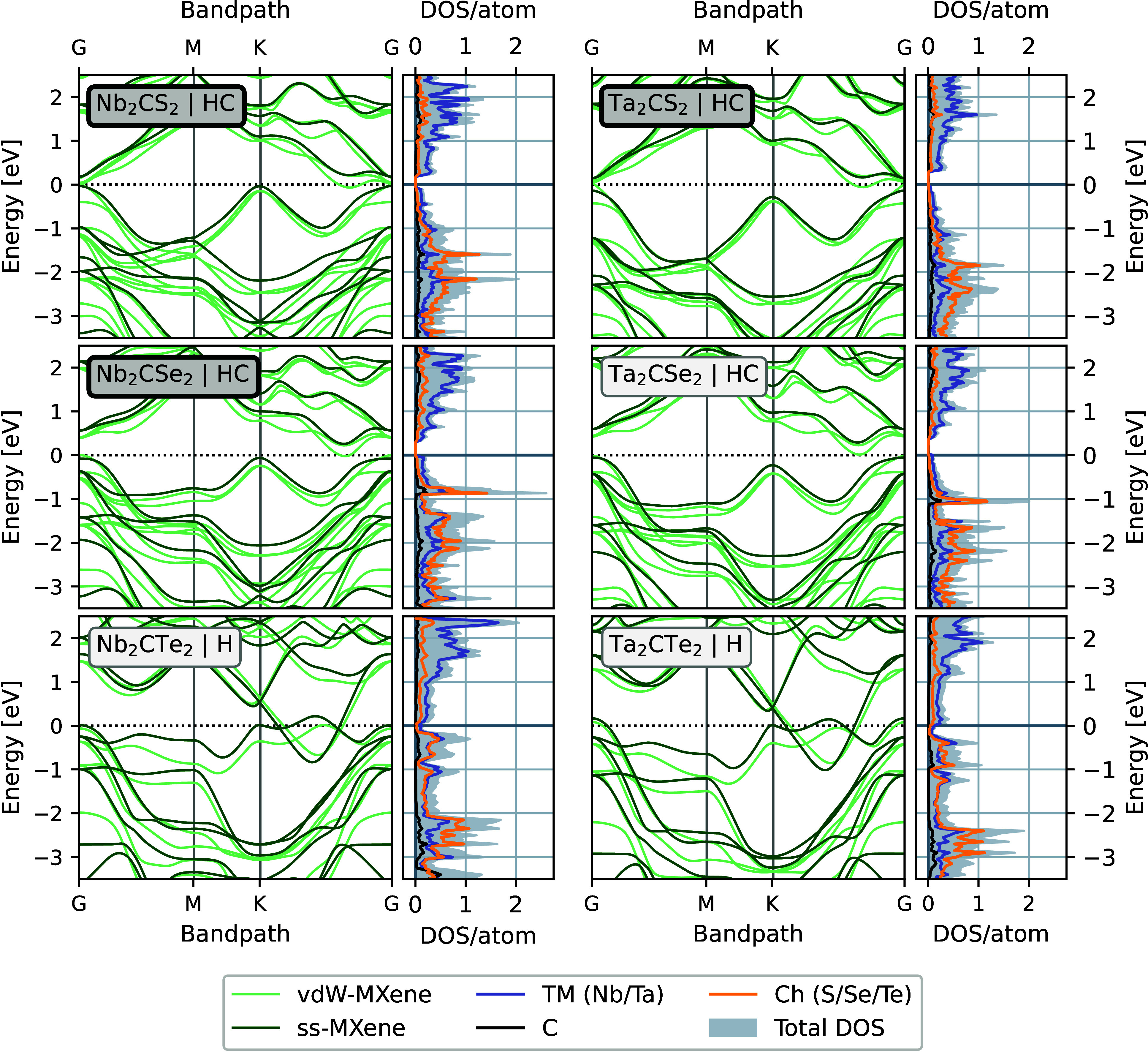
Electronic properties of vdW- and ss-MXenes. Band structures for vdW-MXene are shown in light green and for and ss-MXenes in darker green. The partial DOS refers to the ss-MXene in the respective lowest-energy configuration, as indicated by the letters H or HC next to the chemical formula in each plot. Bold lines around the chemical formula indicate that successful experimental synthesis of the vdW-MXene has been reported.

Out of the remaining six vdW-MXenes predicted to have negative formation enthalpies, the phases with Ch = S or Se prefer the HC-configuration, while those with Ch = Te prefer the H-configuration, as shown in Figure 3a. The electronic band structures for each of the preferred configurations are presented in Figure 5. Here we see that the four compositions that prefer the HC-configuration, shown in Figure 5a–d, are semiconductors with small bandgaps in their ss-MXene forms, while the vdW-MXenes are semimetals in the HC-configuration. The two compositions preferring the H configuration (Figure 5e,f) are both metallic regardless of dimensionality.

**Figure 6:**
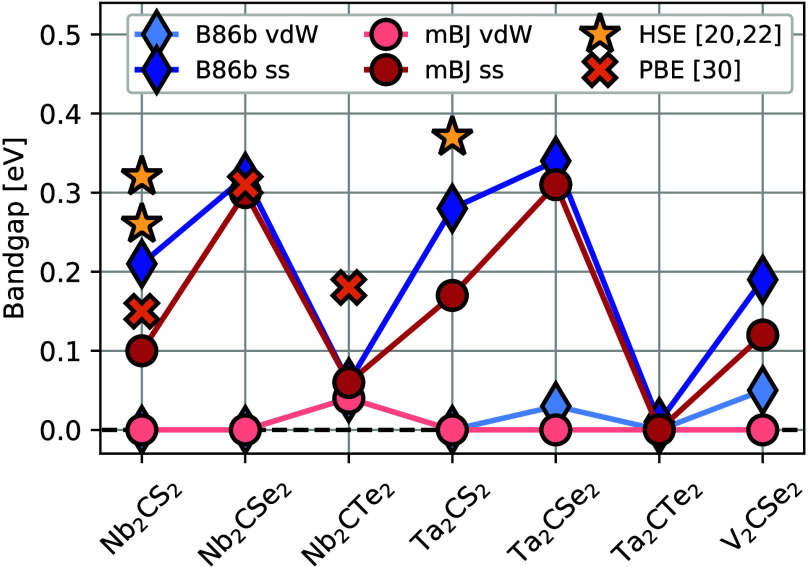
Computed bandgaps. Bandgaps were computed for the HC-configuration of all compositions predicted to have a negative formation enthalpy. Bandgaps for both the vdW-MXene (light colors) and ss-MXene (dark colors) phases are shown, calculated using two different potentials shown in red and blue, respectively. Calculations from previous reports considering ss-MXene are indicated by yellow stars and orange crosses.

Just as for V_2_CSe_2_, the remaining six phases were also studied in their respective nonpreferred configurations out of the H- and HC-configurations. The electronic properties, for the nonpreferred configurations are shown in Figure S5, where again the HC-configuration is shown to give semiconducting or semimetallic properties, and the H-configuration gives metallic properties. The calculated bandgaps for the HC-configurations for all seven compositions can be seen in Figure 6 and are given explicitly in Table S2, for the ss- and vdW-MXene forms using two different potentials as described in the Computational Methods section. The phases including Te display the smallest bandgaps and those containing Se the largest. All ss-MXenes display bandgaps while for the vdW-MXenes the bandgaps are consistently smaller and for several phases close completely.

The bandgaps computed here are systematically smaller than previously reported bandgaps as calculated with a hybrid functional,^21,23^ while they mostly agree well with previous results calculated with the PBE functional.^31^ All previous reports used for comparison consider ss-MXene. Further, we find a larger bandgap using the optB86b-vdW-DF than when using the mBJ functional, in particular for the S-terminated structures and for V_2_CSe_2_.

The ss-Nb_2_CCh_2_ with Ch = S, Se, or Te have all previously been predicted as dynamically stable, and the reported band structures are in excellent agreement with those presented here.^12,21−23,31^ In ref (23) Nb_2_CTe_2_ is determined as dynamically unstable due to small regions of imaginary frequencies around the gamma point. However, this is probably an artifact due to violation of force constant sum rules commonly seen for simulations of phonon dispersions in 2D materials. For the Ta-based chalcogen-terminated MXenes, less computational work has been done previously, and we have only been able to find previous reports on ss-MXene Ta_2_CS_2_.^12,21,22^ In these reports the phase is also identified as dynamically stable, and the presented band structures agree well with ours.^12,21^

## Conclusions

We have performed a computational screening study of the van der Waals bonded (vdW-)MXene phases with the formula M_2_CCh_2_, where M (= Sc, Y, Ti, Zr, Hf, V, Nb, Ta, Mo, or W) is a transition metal and Ch (= S, Se, or Te) is a chalcogen. The study considers the formation enthalpy of the vdW-MXene of each chemical system relative to competing phases as well as dynamical stability through calculation of phonon dispersions. A phase is considered as predicted stable if the formation enthalpy with respect to competing phases is negative, and the phonon frequencies are positive (real). One of the vdW-MXenes previously reported as experimentally realized, Nb_2_CS_2_, has been reported as synthesized via manipulation of the corresponding MAX phase Nb_2_SC. Because of this, the formation enthalpy of M_2_ChC phases has also been screened.

Our study identifies seven vdW-MXenes with negative formation enthalpies that are all dynamically stable in both the vdW- and single sheet (ss-)MXene forms. In addition to verifying the experimentally reported phases Nb_2_CS_2_, Nb_2_CSe_2_, and Ta_2_CS_2_, we identify four additional phases: Ta_2_CSe_2_, Nb_2_CTe_2_, Ta_2_CTe_2_, and V_2_CSe_2_. In particular, V_2_CSe_2_ incorporates an element not previously reported in these phases. The predictions can be applied to experimental realization through traditional metallurgical synthesis, and our results are thus highly relevant for guiding future experimental efforts.

For all structures identified as stable, the delamination energy, electronic band structure, and density of states are presented. The delamination energies are found to lie within a narrow range of 46–57 meV/atom for V_2_CSe_2_ and Nb_2_CTe_2_, indicating that all vdW-MXenes are likely possible to delaminate. In line with previous theoretical reports on the vdW-MXene phases M_2_CCh_2_,^12,16,21−23^ the band structures show that the materials are either metals, semimetals, or small bandgap semiconductors, depending on the specific configuration of the chalcogens and dimensionality. We also identify 15 MAX phases with negative formation enthalpy, out of which 6 are to date experimentally reported.

Although we do not identify any chemical system similar to Nb–C–S in which both the vdW-MXene and corresponding M_2_AX phase are predicted to have formation enthalpies very close to zero, we do identify a number of systems in which the MAX phase is predicted as stable and the vdW-MXene has a positive formation enthalpy below 100 meV/atom. It has been shown that certain MAX phases predicted to have positive formation enthalpies may be synthesized by replacement of the A-layer,^33,34^ and similar methods may be applicable for conversion of M_2_AX to vdW-MXene, rendering the aforementioned phases interesting for further attempts in realizing additional metastable vdW-MXene phases. Besides traditional and chalcogen terminations, MXenes have been reported with a number of other terminations as well, e.g., I and Br.^6^ Although O-terminated vdW-MXenes have been shown previously to exhibit positive formation enthalpies,^36^ the current study clearly shows that this conclusion does not necessarily carry over to all vdW-MXenes, and thus we suggest that further attention may be directed to vdW-MXenes with nontraditional terminations outside of the chalcogen group. It may also be fruitful to go beyond ternary vdW-MXenes and consider quaternary phases by alloying on the M-site.

## Computational Methods

The calculations have been performed within the framework of density functional theory^37,38^ using the Vienna Ab initio Simulation Package (VASP).^39−42^ The cutoff for the plane wave basis set used by VASP was set to 400 eV, and the projected augmented plane-wave (PAW) method was used to model the effect of core electrons.^43,44^ Which electrons were considered as core and valence electrons for each element can be found in Table S1. When possible, the semicore p-electrons for the transition metals were included but the semicore s-electrons were not. For some elements the semicore s-electrons could not be excluded and were thus included for these elements.

The exchange correlation effects were taken into account by combining the correlation from the original van der Waals density functional (vdW-DF)^45^ with optB86b exchange (which is a reoptimization of the B86b exchange^46^), as proposed by Klimeš et al.^47^ This combination of exchange and correlation, commonly denoted as optB86b-vdW-DF, has been shown to give an accurate description of equilibrium geometries for weakly bonded systems,^20^ but also for systems with other binding characteristics.^47^ Additional parameters for the van der Waals contributions were set to the VASP default values. All structures were relaxed until the forces between atoms were smaller than 0.005 eV Å^–1^, and the electronic densities were converged to within 10^–5^ eV/atom. The structures were sampled in a reciprocal space with a *k*-point density of at least 10 points per Å^–1^. An initial assessment of the importance of magnetism in the considered chemical systems indicates insignificant effects on the results, as further discussed in the Supporting Information. Hence, magnetism was not included in the calculations. Electronic bandgaps were evaluated using the modified Becke–Johnsson (mBJ) potential,^48,49^ which has been shown to give good bandgap estimates compared to many other functionals, including hybrid functionals, as well as with the optB86b-vdW-DF scheme.^50^

For each ternary system, all structures found in the Materials Project (MP)^18,19^ database that were within 50 meV/atom of the convex hull as calculated by MP and contained at most 50 atoms per unit cell were considered, and the corresponding convex hull was calculated for each system, using the Python package pymatgen.^51^ The convex hull defines the combination of phases that gives the lowest total energy as a function of stoichiometry. The formation enthalpy per atom Δ*H*_cp_ compared to the set of most competing phases was then evaluated for all considered vdW-MXenes and M_2_AX phases according to
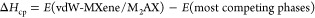
1where *E*(vdW-MXene/M_2_AX) is the energy per atom of the vdW-MXene or M_2_AX phase, and *E*(most competing phases) is the lowest possible energy per atom considering any combination of competing phases at the specific stoichiometry of the vdW-MXene or M_2_AX phase.^52^

If Δ*H*_cp_ is positive, it means that the vdW-MXene or M_2_AX phase has a higher energy than that of the set of most competing phases, rendering it energetically disfavored to the competing phases and thus at best metastable. If, on the other hand, Δ*H*_cp_ is negative, it means that the vdW-MXene or M_2_AX phase has a lower energy than the set of most competing phases, and thus it is energetically favored and predicted thermodynamically stable.

The dynamical stability of vdW-MXenes found to be thermodynamically stable was evaluated using VASP, HiPhive, and phonopy.^53−55^ HiPhive was used to rattle the structures and to fit a force constant potential (FCP) using the forces calculated by VASP, with the Born–Huang sum rules and Huang conditions enforced during fitting. The Perdew–Burke–Ernzerhof (PBE) potential^56^ was used for the VASP calculations. For each phase, the FCP was fitted to forces calculated in 15 rattled structures, and phonopy was successively used to find the phonon band structure given the FCP. The electronic partial and total densities of states were evaluated using LOBSTER.^57−62^

## Supporting Information Available

Details on PAW potentials used for the calculations; descriptions of how the stable termination sites were identified and how different stackings of the ss-MXenes into vdW-MXenes were considered; discussion on the structures found in the current work in relation to previous related work; band structures for vdW- and ss-MXene for the H-/HC-configuration not presented in the main text; phonon dispersion spectra for the H- and HC-configurations for the vdW- and ss-MXene of all compositions predicted to have a negative formation enthalpy; tables over formation energy Δ*H*_cp_ and competing phases for vdW-MXene and M_2_AX of each considered composition (PDF)
