# Supplementary material for: Computational Screening of Chalcogen-Terminated Inherent Multilayer MXenes and M2AX Precursors
Source: Inorg Chem. 2024 Aug 26;63(36):16645–54. doi: 10.1021/acs.inorgchem.4c01690 (PMC11388472; doi:10.1021/acs.inorgchem.4c01690)
Supplement: Supplementary file 1 — ic4c01690_si_001.pdf [file ic4c01690_si_001.pdf]

Supporting Information: Computational Screening of Chalcogen-Terminated Inherent Multilayer MXenes and M_2AX Precursors

Pernilla Helmer, Jonas Björk, and Johanna Rosen*

Department of Physics, Chemistry and Biology, Linköping University, Linköping

E-mail: johanna.rosen@liu.se

Computational Details - PAW Potentials

Table S1 shows which PAW-potentials were used for each element, and the electrons explicitly considered in the simulations are specified.

Termination Site Screening

Figure S1 shows the result of the site screening for 6 by 6 termination sites in the unit cell for the four selected structures Nb_2CS_2, Nb_2CTe_2, V_2CSe_2 and Ta_2CS_2. The position of each data point indicates the starting position of the terminations before relaxation. The color and marker of each data point indicate the final position of the terminations. The yellow or green data points without a marker, corresponding to termination at the M-site, also indicate the position of the M-site in the unit cell. The center data point of the areas marked by crosses or circles coincide with the H- and C-sites, respectively. Without exception, The C- and H-sites are stable with 10 and 25 of the 36 configurations relaxed into each site

Table S1: Electrons considered as valance electrons for each element. For the transitions metals, the semi-core electrons from the 3p/4p/5p orbitals were included for accuracy, while the 3s/4s/5s electrons were excluded if possible due to computational considerations. For the transition metals Y and Zr, the only PAW-potential available included both p- and s-semi core electrons. For Sc, only PAW potentials including both p- and s-semi core electrons or no semi core electrons were available, in which case the potential including more electrons was chosen.

Element	POTCAR ending	Valance electrons
S		3p 3s
Se		4p 4s
Te		5p 5s
C		2p 2s
Sc	_sv[a]	4s 3d 3p 3s
Ti	_pv	4s 3d 3p
V	_pv	4s 3d 3p
Y	_sv[a]	5s 4d 4p 4s
Zr	_sv[a]	5s 4d 4p 4s
Nb	_pv	5s 4d 4p
Mo	_pv	5s 4d 4p
Hf	_pv	6s 5d 5p
Ta	_pv	6s 5d 5p
W	_pv	6s 5d 5p

[a] No _pv potential available.

respectively. The H-site was energetically preferred for all compositions, by between 20-65 meV/atom. The M-site is considerably higher in energy for all four probed structures, and only stable when constrained by symmetry. When not constrained, also these configurations relaxed into one of the two stable configurations. Hence, the M-site was not considered further as a possible termination site.

The preference for the C- or H-site may be understood through the higher electronegativity of the chalcogen species Ch compared to that of the transition metal, thus maximizing the electrostatic attraction between the termination and the pristine MXene at these sites. C also has a higher electronegativity than the transition metal, so that the C-atoms are also likely to have an excess of electrons. Thus, the Ch- and C-atoms would both be negatively charged and repel each other, giving an intuitive explanation to why the H-site is preferred

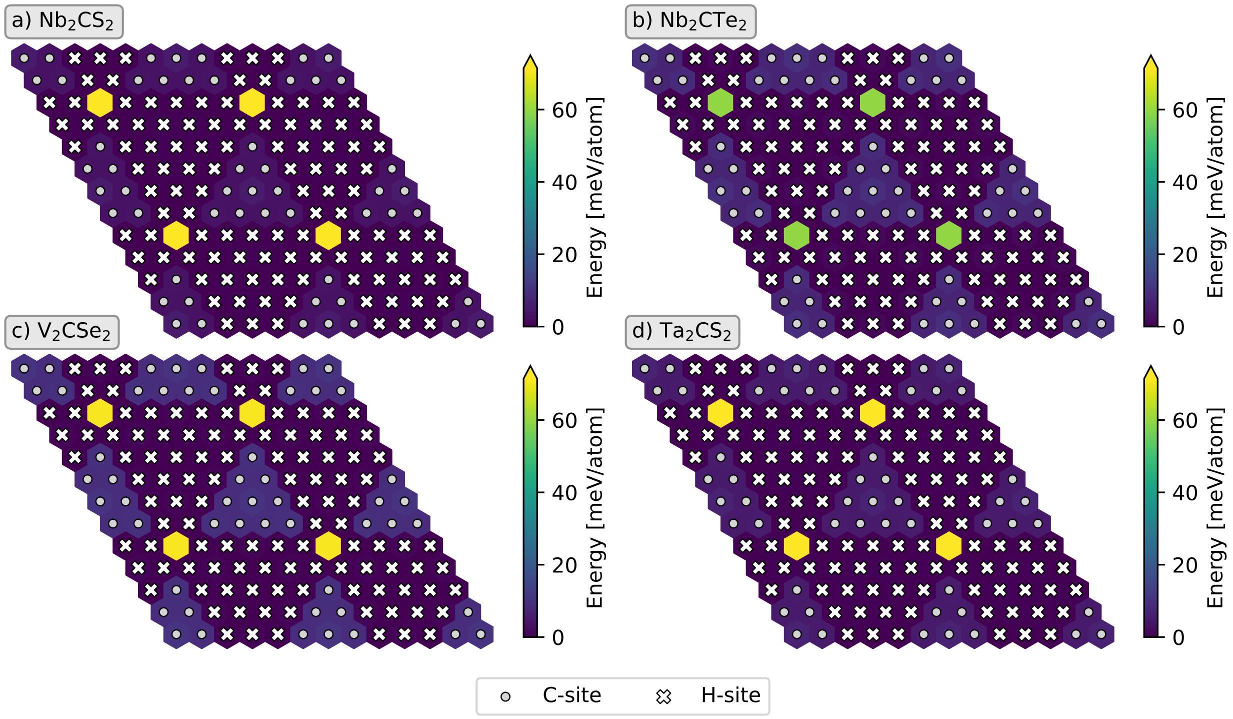

Figure S1: Site screening of the Ch-terminations for a selected set of four vdW-MXenes. The MXene-surface was divided into 6 by 6 sites at which the Ch-terminations were placed symmetrically on each side of the MXene. The whole structure was then allowed to relax. The resulting structures were categorized according to distance between the Ch-terminations and the central C-atom of the unit cell. For each of the four considered structures, the Ch-terminations were found in one out of three final positions: C-site (circle), H-site (cross) or M-site (no marker, yellow or green colored hexagon). As is clearly seen, the M-site is by far highest in energy, and was only stable under symmetry constraints, while the H-site is consistently preferred. The zero point is taken as the adsorption energy at the H-site for each composition.

to the C-site.

Stacking Schemes

Having identified the likely termination configurations, as described in the main text, different vdW-MXene structures were constructed by stacking the terminated MXene sheets in different ways. Since the possibilities for stacking sequences are innumerous, we chose to first consider only the H-configuration for the terminations, since the initial site screening of Figure S1 indicates that the H-site is energetically preferred for all four screened structures.

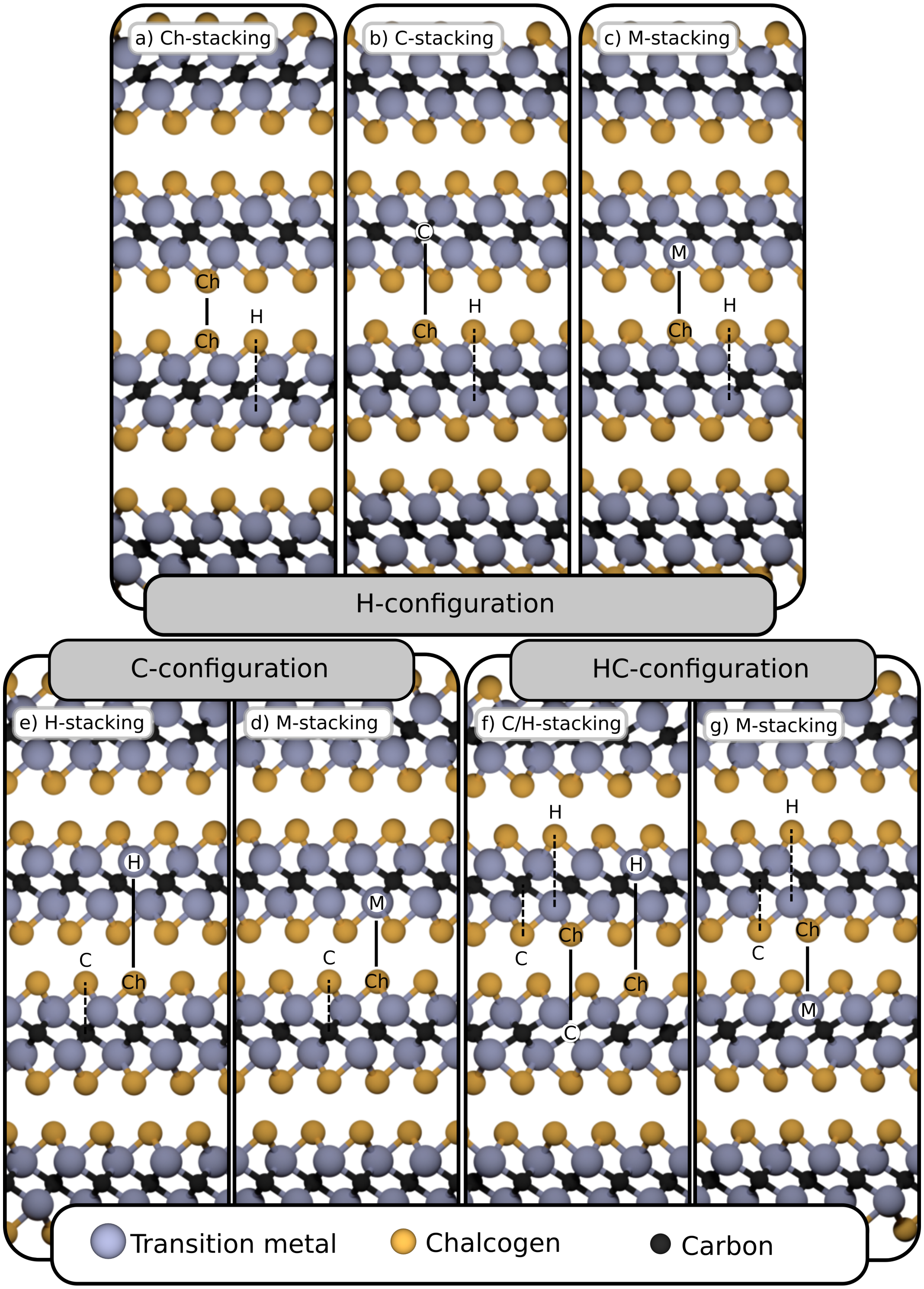

Figure S2: Schematic stacking configurations of Ch-terminated MXene. Panels a-c) show the H-configuration in Ch-stacking (a), C-stacking (b) and-M stacking (c). Panels d-e) show the C-configuration in H-stacking (d) and M-stacking (e). Panels f-g) show the HC-configuration in the C/H-stacking (f) and M-stacking (g).

Full H-site termination is also in agreement with experimental reports.[S1–S4] Three different stacking sequences were considered, as displayed in Figure S2a-c). In a), the Ch-terminations are stacked on top of each other between layers (Ch-stacking), in b), Ch of one layer are aligned to the C-sites of the adjacent layer (C-stacking), and in c) Ch are aligned with the M-sites of the adjacent layer (M-stacking). The resulting vdW-MXene structures were fully relaxed for all stoichiometries M_2CCh_2.

In general, most stoichiometries preferred the Ch-terminations to face the M-site of the adjacent layer, *i.e.*M-stacking, as depicted in Figure S2c). In particular, this is true for the vdW-MXenes that in the present work were found to be thermodynamically stable. This is in agreement with experimental results for vdW-Ta_2CS_2, where two different stackings have been identified.[S1] The phase formed at high temperatures was found to prefer the 3s stacking, defined in ref. S1, which corresponds to C-stacking in the present work. However, upon grinding at lower temperatures the structure was found to transform to the 1s stacking, again defined in ref. S1, which corresponds to M-stacking in this work, and upon successive heating the structure was found to transform into a more complicated layered structure suggested to contain significant fractions of the 3s/C-stacking. Experimental work on vdW-Nb_2CS_2 reports M-stacking for this phase, which was altered to C-stacking upon mechanical disturbance.[S4] Ref. S2 instead reports stacking disorder for vdW-Nb_2CS_2.

Out of the 30 different stoichiometries, 20 prefer M-stacking for the H-configuration, while 7 prefer C-stacking. The three remaining stoichiometries: Sc_2CS_2, Y_2CS_2 and Y_2CSe_2, prefer Ch-stacking. Upon closer inspection of the structures, it was seen that the Ch-Ch distance between layers in the structures which preferred Ch-stacking was considerably shorter than in structures preferring M- or C-stacking. This implies relatively strong bonding between the Ch terminations in structures preferring Ch-stacking, as opposed to the expected weak inter-layer bonding. Additionally, in all of these three systems (Sc_2CS_2, Y_2CS_2 and Y_2CSe_2), the considered phases had positive formation enthalpies of more than 500 meV/atom, rendering them of low interest for further consideration since they are unlikely to be synthesizable. For

the 27 stoichiometries preferring either the H- or C-stackings, the Ch-stacking was consistently highest in energy out of the three considered stacking schemes. Hence, Ch-stacking was not considered further.

Disregarding the three systems preferring Ch-stacking for the H-configuration, all S- and Se-terminated structures with metals from group III and IV (Sc, Y, Ti, Hf and Zr) preferred C-stacking, while the remaining structures, *i.e.*, all Te-terminated structures and structures with metals from groups V and VI (V, Nb, Ta, Mo and W), preferred M-stacking. The differences between C- and M-stackings were within 15 meV/atom for all stoichiometries, and within 10 meV/atom for most. Disregarding the stoichiometries where the Ch-stacking is the most beneficial (and seemingly covalent in nature), the difference between the preferred stacking and the Ch-stacking was at most 21 meV/atom. This clearly shows that the stacking sequence is less important than the termination site.

Next to be considered is the C-configuration. For this configuration, two stacking schemes were considered: One where the Ch-terminations aligned with the H-site, as indicated in Figure 1b), as well as the already discussed M-stacking. The former is in the following referred to as the H-stacking, and is fully depicted for the C-configuration in Figure S2d), while the M-stacked C-configuration is shown in Figure S2e). Out of the 30 stoichiometries, 21 preferred M-stacking, while 9 preferred H-stacking. For all stoichiometries with Ch=Te or with a transition metal from groups V and VI (*i.e.*V, Nb, Ta, Mo or W) the M-stacking was preferred, while for stoichiometries with Ch=S or Se and a transition metal from groups III or IV (i .e. Sc, Y , Ti, Zr or Hf) H-stacking was preferred, with the exception for Y_2CS_2 which preferred M-stacking. However, the difference between the M- and H-stacking for the stoichiometries containing Y were only 2 meV/atom independent on chalcogen species, which can not be considered significant in this context. Including all stoichiometries, the difference between the two stackings ranged between 1 and 34 meV/atom, with the largest differences for Ch = S or Se in Zr_2CCh_2 (17 resp. 34 meV/atom), Ti_2CCh_2 (33 resp. 7 meV/atom) and Hf_2CCh_2 (20 resp. 24 meV/atom).

Finally, also the HC-configuration was considered, including M-stacking and a mixed C/H-stacking shown in Figures S2g) and f) respectively. Since the terminations populate the C-sites on one side of the ss-MXene and the H-sites on the other side, pure C- or H-stacking is not possible, and, as argued for earlier, Ch-stacking was not considered. All phases predicted stable in this work preferred M-stacking, as do all but Sc_2CS_2, Y_2CSe_2 and M_2CCh_2 for M=Ti, Zr or Hf and Ch= S or Se. Considering all of the studied chemical systems, the M- and H/C-stackings are within less than 1 to at most 14 meV/atom.

History and Discussion on Screening Results

We will continue the short discussion given in the main text on what has been done in previous studies on the chalcogen terminated vdW-MXenes and M_2AX phases with A=S, Se or Te, to put our results in further perspective. In Figure 2, phases which has previously been experimentally reported are implied by black, solid circles, while theoretical studies predicting a phase stable from some perspective are indicated by grey and black dashed circles. More details are given in the main text. The most competing phases for each of the herein studied phases are listed in Tables S3-S8.

Our results for the MAX phases are mostly in agreement with previous work.[S5] Discrepancies are found for Nb_2SC, V_2SC, Sc_2SeC and Y_2SeC. Nb_2SC has been previously reported as having a small positive formation enthalpy, while we predict the formation enthalpy to be barely negative. On the other hand, V_2SC is predicted stable in previous work, while we find a positive formation enthalpy of 8 meV/atom. These are not very grave disagreements, and ours are in better agreement with experimental reports, which reports Nb_2SC as readily synthesizeable.[S6] Sc_2SeC and Y_2SeC, which are here predicted stable, have been predicted as unstable in a previous report. Since previous work does not specify which competing phases have been considered, it is difficult to deduce what might cause this disagreement. Besides Nb_2SC, both the present and previous theoretical work are in agreement with experimental

reports, identifying all other experimentally reported MAX phases as stable. The phase Ti_2TeC, which is here predicted to have a small positive formation energy of 16 meV/atom, is marked with a grey dashed circle to indicate that also in previous studies this phase has been found to be near stable.

Out of the vdW-MXenes, four have been predicted stable in previous work with Ch=S, out of which we identify two.[S7] The discrepancies can be attributed to the set of competing phases in previous work not including Hf_2SC (MAX phase), HfS_2 (Materials Project id mp-985829), V_3S_4 (mp-799) and V_6C_5 (mp-28731), found in this work to be among the most competing phases for the respective vdW-MXene. The fact that we can show full agreement with experimentally identified phases is reassuring and speaks in favor of our results. Although, as mentioned earlier one of the phases predicted stable in this work, Nb_2CS_2, has to date not been reported as synthesized directly through solid state synthesis, implying it is only metastable. This is however not necessarily indicative of an inconsistency with experiments, since synthesis conditions are in general much more complex than computational conditions, and while the formation enthalpy is a good indicator of thermodynamic stability, it is not perfect and should be used as a guide for experimental efforts.

In addition, theoretical studies have been conducted on several vdW-MXene or ss-MXene phases within the here considered chemical systems where a rigorous stability analysis has not been included.[S8–S16] These are marked by dashed grey circles in Figure 2. By rigorous stability analysis, we mean that the termodynamical stability ofthe vdW-MXene has been assessed through evaluation of ΔH_{cp}, in addition to consideration of the dynamical stability. Among the M_2CS_2 phases, Sc_2CS_2 and Y_2CS_2 ss-MXenes have been investigated for use within gas sensing,[S10] although in a skewed structure not considered in this work. Previous work, in which the skewed structure for Sc_2CS_2 and Y_2CS_2 ss-MXene was identified, did not include a stability analysis from the perspective of formation enthalpy, but focused merely on dynamical stability though calculation of phonon spectra and molecular dynamics simulations.[S9] Since we in the present study found the investigated (non-skewed) vdW-

MXene phase for Sc_2CS_2 and Y_2CS_2 to be thermodynamically unstable by several hundred meV/atom, we chose to not include the skewed structure in our study.

To the best of our knowledge, no reports of systematic thermodynamic screening have been made on the vdW-MXenes in the M-C-Se and M-C-Te chemical systems. Earlier stability analysis made on MXene structures within these chemical systems have been limited to the ss-MXene phase and the dynamical stability through calculation of phonon dispersions and molecular dynamic simulations. In some cases, the studies have included analysis of the formation energy with respect to unterminated MXene and the respective ground state of the chalcogen in question.[S15] However, since an unterminated MXene has yet to be reported in the delaminated state, we argue this kind of formation energy analysis is of limited predictive value.

When it comes to experimental results for the vdW-MXenes, Nb_2CS_2 and Ta_2CS_2 has been previously reported multiple times,[S1–S4] and Nb_2CSe_2 vdW-MXene has been reported once.[S17] These three phases have been marked by black circles in Figure 2, to indicate that they have been reported as experimentally realized. Ta_2CSe_2 is alluded to in the literature, but we have been unable to find a reliable source for the synthesis of this phase.[S18] Ta_2CSe_2 has thus been left unmarked in Figure 2. The three phases Nb_2CTe_2, Ta_2CTe_2 and V_2CSe_2 have not previously been reported experimentally. Additionally, Ti_2CCh_x has been reported via termination substitution.[S19] However, the termination position and concentration was not thoroughly established in the previous report, and the suggested termination positions and concentration agrees poorly with the present work. Therefore we have not marked these phases as experimentally reported in Figure 2, but mention them here for completeness.

We have also compared our results for the termination sites with previous theoretical and experimental studies. Experimental reports on Nb_2CS_2, Ta_2CS_2 and Nb_2CSe_2 all give the H-configuration as the experimental structure.[S1–S4] Previous theoretical reports have, just as we do, identified the HC-configuration as the most energetically favored for Nb_2CS_2, Nb_2CSe_2 and Ta_2CS_2,[S7,S9,S15,S20] while Nb_2CTe_2 and V_2CSe_2 prefer the H-configuration.[S15,S20] For the

two phases Ta_2CSe_2 and Ta_2CTe_2, we have found no previous theoretical work. In addition, several theoretical studies focused on properties for potential applications have assumed a H-configuration structure also for the structures predicted to prefer the HC-configuration, in line with experimental reports.[S3,S12] Measurements on superconductivity of Nb_2CS_2 imply that the as-synthesized vdW-MXene is indeed a conductor, indicating a discrepancy between experiments and theoretical results for this phase.[S3,S4]

Additional Comments to Screening Results

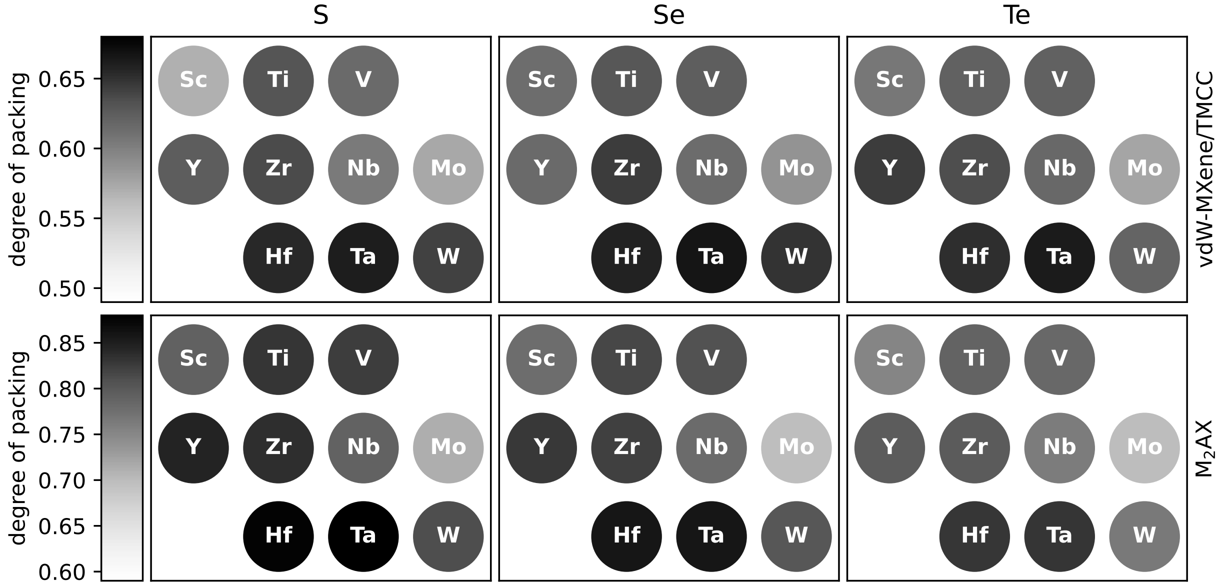

Figure S3: Degree of packing ρ for each of the considered vdW-MXene and MAX-phases, as defined in equation S1.

As noted in the main text, a trend of the MAX-phases being more stable for smaller chalcogen size may be observed. It is far from trivial to analyze why this is, but a correlation with degree of packing can be identified. The degree of packing ρ, is here defined as the total volume of the atoms in the unit cell V_{atoms}, divided by the unit cell volume $V_{u.c.}$. I.e.,

$$\rho = \frac{V_{atoms}}{V_{u.c.}}. \tag{S1}$$

The covalent radii as given in the Atomic Simulation Environment data module have been used to evaluate V_{atoms}. Figure S3 shows the degree of packing as attained though this method. It can be seen that within any set of MAX-phases $\{M_2CS, M_2CSe, M_2CTe\}$, the degree of packing reduces as the size of the chalcogen species increases, correlating with the decrease in stability as the size of the chalcogen species increases. It should be noted, however, that although this correlation exists for the MAX-phases, the degree of packing can not be used to predict the thermodynamical stability, since no cut-off value separating stable from unstable phases can be identified.

Magnetism has for the most part not been included in this study, since none of the traditionally magnetic transition metals was considered. Never the less, we have considered the possible effect of magnetism in the V-C-Se-system. We chose this specific system both because the vdW-MXene V_2CSe_2 in this system is predicted stable, and because V is the transition metal out of the considered ones which is most likely to exhibit magnetic properties. One of the competing phases, VSe_2, is magnetically ordered according to Materials Project. Hence, we included a magnetic version of this phase in the set of competing phases, thus increasing the formation energy from -43 meV to -42 meV per atom. Thus is a non-significant difference smaller than the expected accuracy for these calculations. We thus conclude that although some of these material may display magnetic ordering, the effect on the formation enthalpy is insignificant and does not alter the predictions.

Properties

Figure S4 shows electronic properties and phonon dispersion for V_2CSe_2 in the HC-configuration, which is disfavored to the H-configurations by 18 meV/atom. The structure is dynamically stable in both its vdW and single sheet form, indicated by the lack of negative (imaginary) frequencies in the phonon spectra. It also displays a small electronic bandgap.

Figure S5 shows the electronic band structure of the energetically less preferred configu-

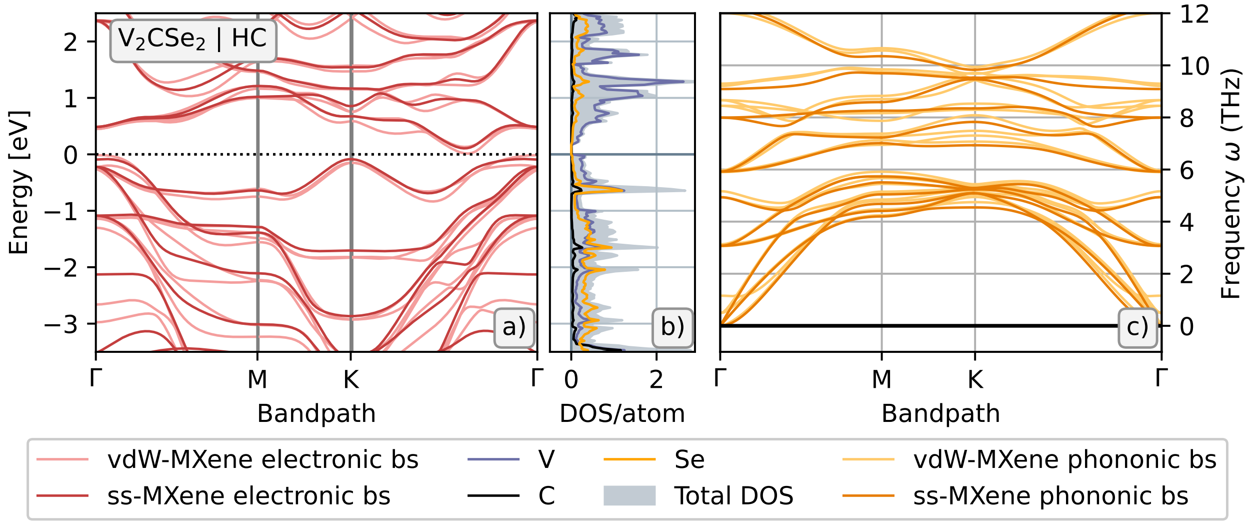

Figure S4: Properties for V_2CSe_2. a) Electronic band structure, b) DOS and c) phonon dispersion for V_2CSe_2 in the HC-configuration. Lighter colors show data for the vdW-MXene and darker colors for the ss-MXene. The DOS refers to ss-V_2CSe_2

ration out of the H- and HC-configurations for vdW- and ss-MXene with M=Nb or Ta and Ch= S, Se or Te. It can be seen that the H-configuration is metallic and the HC-configuration is semiconducting or semimetallic. Figure S7 shows the respective phonon dispersions, indicating dynamical stability of all phases in both their vdW and single sheet forms. Figure S6 shows the phonon dispersion of the energetically preferred configurations of each of the stoichiometries with M=Nb or Ta and Ch= S, Se or Te, for which the electronic properties are shown in Figure 5 of the main text, also here indicating dynamical stability.

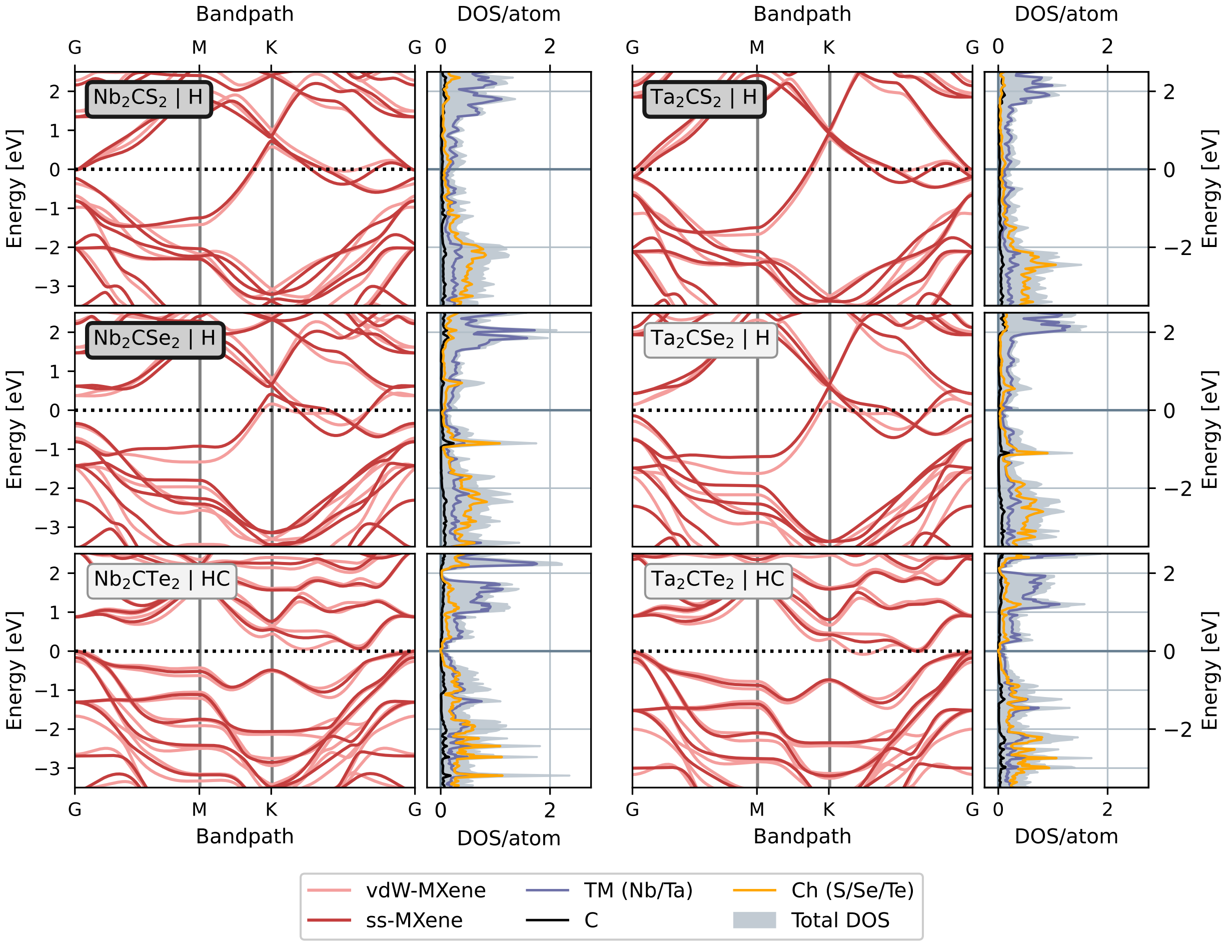

Figure S5: Electronic properties for vdW- and ss-MXenes. Electronic band structures and DOS for the less energetically beneficial configuration out of the H- and HC-configurations for the six vdW-MXenes predicted stable where M=Nb or Ta. Light red indicates vdW-MXene and the darker red indicates ss-MXene. The configuration is indicated by the letters H or HC next to respective chemical formula, and the dark border around the chemical formula imply experimental realization of the vdW-MXene. DOS refer to ss-MXenes.

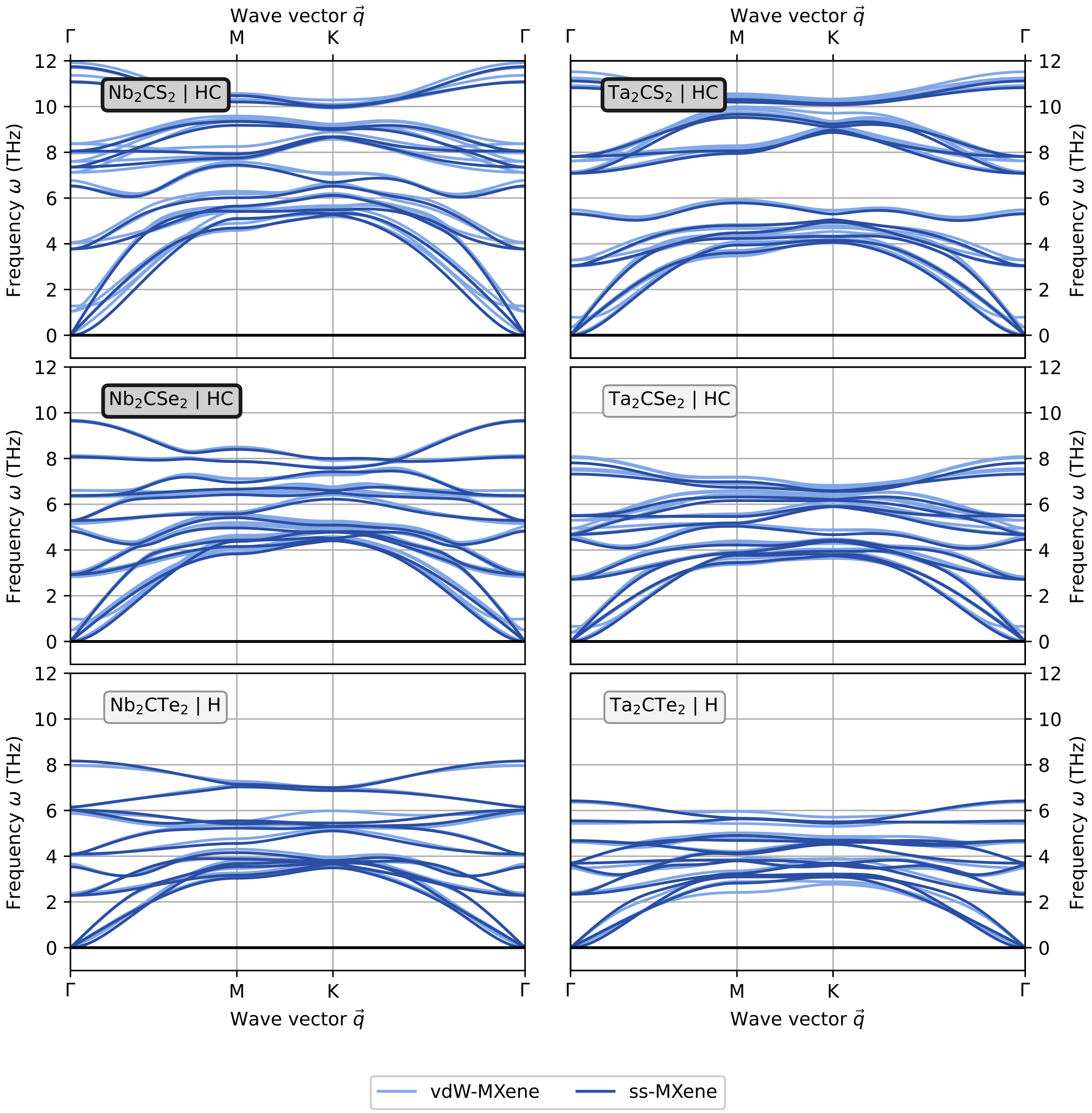

Figure S6: Phonon dispersions for the vdW-MXenes and ss-MXenes with M=Nb or Ta, in their respective preferred configuration out of the H- and HC-configurations. Lighter color refers to vdW-MXene and darker color refers to ss-MXene. The configuration is indicated by the letters H or HC next to respective chemical formula, and the dark border around the chemical formula imply experimental realization of the vdW-MXene.

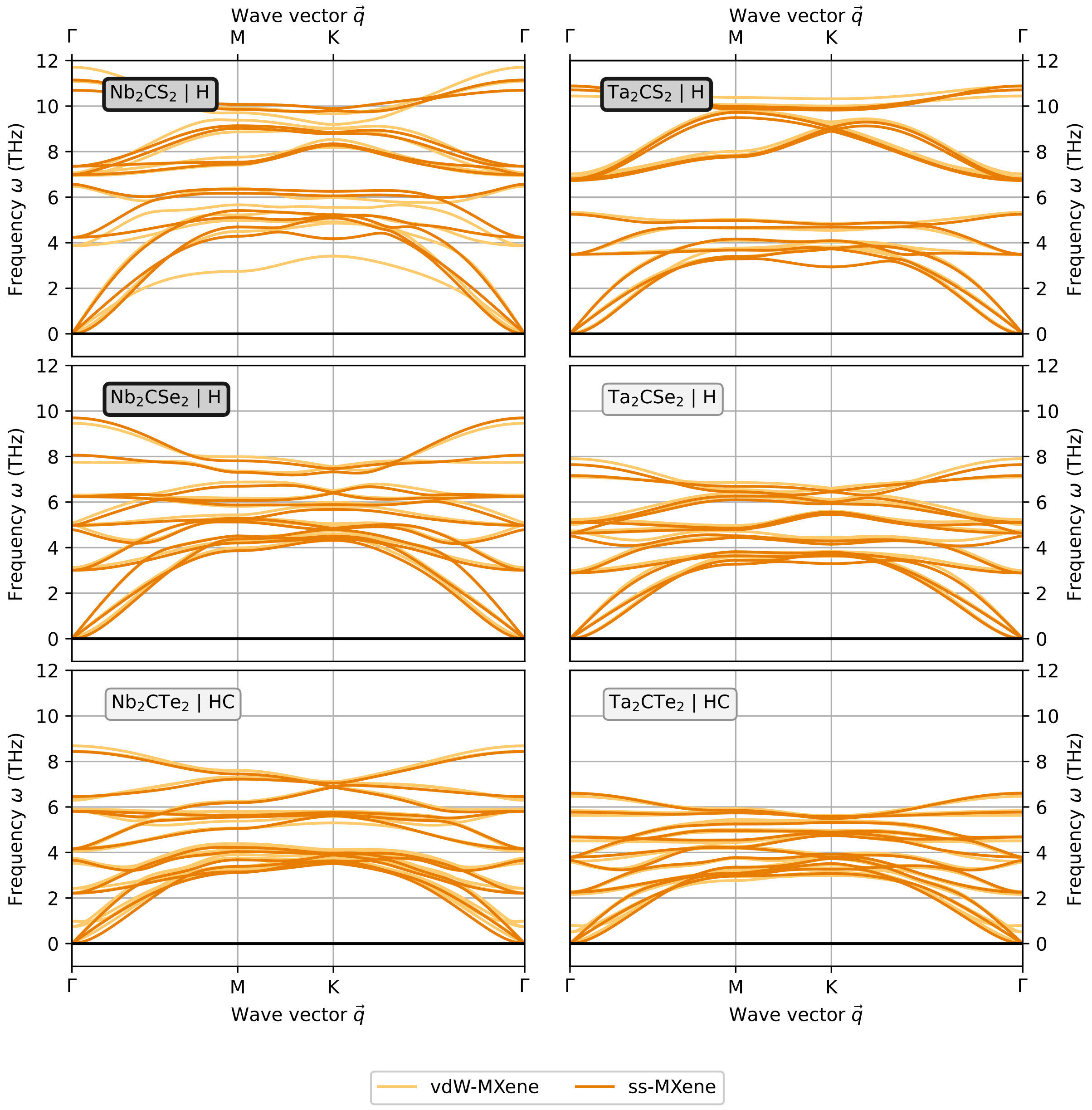

Figure S7: Phonon dispersions for the vdW-MXenes and ss-MXenes with M=Nb or Ta, in their respective non-preferred configuration out of the H- and HC-configurations. Lighter color refers to vdW-MXene and darker color refers to ss-MXene. The configuration is indicated by the letters H or HC next to respective chemical formula, and the dark border around the chemical formula imply experimental realization of the vdW-MXene.

Table S2: Bandgaps for the HC-configurations of the vdW-M_2CCh_2 predicted to have $\Delta H_{cp} < 0$, and the corresponding ss-M_2CCh_2.

	optB86b-vdW-DF[eV]		mBJ [eV]		
composition	vdW	ss	vdW	ss	previous [eV]
Nb_2CS_2	0.0	0.21	0.0	0.10	0.26,[S9] 0.15,[S14] 0.32[S20]
Nb_2CSe_2	0.0	0.32	0.0	0.30	0.31[S14]
Nb_2CTe_2	0.04	0.06	0.04	0.06	0.18[S14]
Ta_2CS_2	0.0	0.28	0.0	0.17	0.37[S9]
Ta_2CSe_2	0.03	0.34	0.0	0.31	
Ta_2CTe_2	0.0	0.01	0.0	0.0	
V_2CSe_2	0.05	0.19	0.0	0.12	

[S9,S20] as calcultated with HSE06,[S14] as calculated with PBE

Tables over competing phases

Formation enthalpies are given with respect to elementary, binary and ternary phases, *i.e.*, the vdW-MXene phase is considered in the formation enthalpy for a MAX phase, and vice versa. All competing phases are taken from Materials Project (MP),[S21] with the addition of the M_2AX phases and M_2CCh_2-phases studied in the present work. No ternary phase was found at MP within these systems that was not either a vdW-MXene on the form M_2CCh_2 or a M_2AX phase. Phases without an MP-id are either vdW-MXene or MAX phases constructed in this work.

The tables may be read as follows: the first column indicates the chemical formula of the respective studied vdW-MXene or M_2AX phase. The second column indicates the formation enthalpy ΔH_{cp} of the phase, *i.e.*, the number encoded by the color in Figure 2. The third column indicates the chemical formulas of the set of most competing phases (MCPs) to the vdW-MXene or M_2AX phase. The fourth column is the fraction of each MCP needed to make up the correct stoichiometry. The fifth column gives the energy per atom as calculated for the purpose of this screening study. And finally, the sixth column gives the MP-id. To evaluate ΔH_{cp} for Sc_2CS_2, as an example, we perform the following computation:

$$\begin{aligned}\Delta H_{cp}(\mathrm{Sc_2CS_2}) &= E_{\mathrm{Sc_2CS_2}} - (f_{\mathrm{C}} * E_{\mathrm{C}} + f_{\mathrm{ScC}} * E_{\mathrm{ScC}}) \\ &= -5.661\ \mathrm{eV} - (0.2 * (-9.067\ \mathrm{eV}) + 0.8 * (-5.459\ \mathrm{eV})) \\ &= 0.519\ \mathrm{eV}.\end{aligned}$$

Here E_{A} is the energy (in eV/atom) of phase A, listed in column 5, and f_{A} is the fraction of phase A needed to make up the correct stoichiometry, listed in column 4. For the specific example of Sc_2CS_2, we see that

$$0.2 * 1\,\mathrm{C} + 0.8 * \left(\frac{1}{2}\,\mathrm{Sc} + \frac{1}{2}\,\mathrm{S}\right) = 0.2\ \mathrm{C}{:}0.4\ \mathrm{Sc}{:}0.4\ \mathrm{S},$$

which is indeed the stoichiometry of Sc_2CS_2. The factor 1/2 for ScS comes from this phase having *two* atoms in the chemical formula, and the energies being given in eV/atom, not per formula unit.

Table S3: Competing phases for vdW-MXenes with S-terminations.

Phase	ΔH_{cp} [eV/atom]	comp. phases	fraction	Energy [eV/atom]	MP-id
Sc_2CS_2	0.519			-5.661	
		C	0.200000	-9.067	48
		ScS	0.800000	-5.459	1476
Y_2CS_2	0.641			-5.841	
		C	0.200000	-9.067	48
		YS	0.800000	-5.836	1534
Ti_2CS_2	0.161			-6.151	
		Ti_2CS	0.400000	-6.970	
		Ti_2S_3	0.500000	-5.235	1101099
		C	0.100000	-9.067	48
Zr_2CS_2	0.161			-6.857	
		Zr_2CS	0.533333	-7.806	5025
		ZrS_2	0.400000	-5.625	1186
		C	0.066667	-9.067	48
Hf_2CS_2	0.106			-7.563	
		Hf_2CS	0.533333	-8.592	
		HfS_2	0.400000	-6.205	985829
		C	0.066667	-9.067	48
V_2CS_2	0.048			-6.436	
		V_3S_4	0.700000	-5.531	799
		V_6C_5	0.183333	-8.475	28731
		C	0.116667	-9.067	48
Nb_2CS_2	-0.001			-7.164	
		Nb_3S_5	0.640000	-5.983	32983
		Nb_6C_5	0.293333	-9.307	2760
		C	0.066667	-9.067	48
Ta_2CS_2	-0.064			-8.007	
		$Ta_{27}S_{50}$	0.616000	-6.474	530018
		TaC	0.368000	-10.353	1086
		C	0.016000	-9.067	48
Mo_2CS_2	0.074			-7.044	
		MoC	0.400000	-9.236	2305
		MoS_2	0.600000	-5.708	2815
W_2CS_2	0.131			-7.885	
		WC	0.400000	-10.414	1894
		WS_2	0.600000	-6.418	224

Table S4: Competing phases for vdW-MXenes with Se-terminations.

Phase	ΔH_{cp} [eV/atom]	comp. phases	fraction	Energy [eV/atom]	MP-id
Sc_2CSe_2	0.519			-5.177	
		Sc_2CSe	0.400000	-6.171	
		Sc_2Se_3	0.500000	-4.641	1205354
		C	0.100000	-9.067	48
Y_2CSe_2	0.576			-5.405	
		C	0.200000	-9.067	48
		YSe	0.800000	-5.209	2637
Ti_2CSe_2	0.085			-5.727	
		Ti_2CSe	0.533333	-6.588	
		$TiSe_2$	0.400000	-4.235	2194
		C	0.066667	-9.067	48
Zr_2CSe_2	0.125			-6.437	
		Zr_2CSe	0.533333	-7.468	
		$ZrSe_2$	0.400000	-4.936	2076
		C	0.066667	-9.067	48
Hf_2CSe_2	0.098			-7.103	
		HfC	0.400000	-9.802	21075
		$HfSe_2$	0.600000	-5.467	985831
V_2CSe_2	-0.043			-6.033	
		V_6C_5	0.366667	-8.475	28731
		C	0.033333	-9.067	48
		VSe_2	0.600000	-4.300	694
Nb_2CSe_2	-0.073			-6.763	
		Nb_6C_5	0.366667	-9.307	2760
		C	0.033333	-9.067	48
		$NbSe_2$	0.600000	-4.958	2207
Ta_2CSe_2	-0.084			-7.562	
		TaC	0.400000	-10.353	1086
		$TaSe_2$	0.600000	-5.561	680184
Mo_2CSe_2	0.110			-6.587	
		MoC	0.400000	-9.236	2305
		$MoSe_2$	0.600000	-5.005	1634
W_2CSe_2	0.181			-7.374	
		WC	0.400000	-10.414	1894
		WSe_2	0.600000	-5.648	1821

Table S5: Competing phases for vdW-MXenes with Te-terminations.

Phase	ΔH_{cp} [eV/atom]	comp. phases	fraction	Energy [eV/atom]	MP-id
Sc_2Te_2C	0.229			-4.833	
		Sc_2TeC	0.400000	-5.687	
		Sc_2Te_3	0.500000	-3.761	685069
		C	0.100000	-9.067	48
Y_2Te_2C	0.419			-4.952	
		Y_2TeC	0.400000	-5.965	
		Y_2Te_3	0.500000	-4.157	1189791
		C	0.100000	-9.067	48
Ti_2Te_2C	0.088			-5.197	
		$TiTe_2$	0.600000	-3.383	1907
		TiC	0.400000	-8.138	631
Zr_2Te_2C	0.072			-5.916	
		$ZrTe_2$	0.600000	-4.059	1018107
		ZrC	0.400000	-8.883	2795
Hf_2Te_2C	0.095			-6.538	
		$HfTe_2$	0.600000	-4.519	32887
		HfC	0.400000	-9.802	21075
V_2Te_2C	0.040			-5.476	
		VTe_2	0.480000	-3.501	11687
		V_6C_5	0.440000	-8.475	28731
		Te	0.080000	-1.331	567313
Nb_2Te_2C	-0.032			-6.232	
		Nb_6C_5	0.366667	-9.307	2760
		$NbTe_2$	0.600000	-4.141	11675
		C	0.033333	-9.067	48
Ta_2Te_2C	-0.024			-6.974	
		$TaTe_2$	0.600000	-4.681	1967
		TaC	0.400000	-10.353	1086
Te_2Mo_2C	0.129			-6.067	
		MoC	0.400000	-9.236	2305
		Te_2Mo	0.600000	-4.169	602
Te_2W_2C	0.206			-6.815	
		WC	0.400000	-10.414	1894
		Te_2W	0.600000	-4.759	22693

Table S6: Competing phases for M_2AX phases with S as A-element.

Phase	ΔH_{cp} [eV/atom]	comp. phases	fraction	Energy [eV/atom]	MP-id
Sc_2CS	-0.162			-6.510	
		$Sc_{15}C_{19}$	0.274194	-7.480	15957
		Sc_4C_3	0.225806	-6.938	15661
		ScS	0.500000	-5.459	1476
Y_2CS	-0.119			-6.703	
		Y_2C	0.125000	-6.558	1334
		Y_4C_5	0.375000	-7.589	9459
		YS	0.500000	-5.836	1534
Ti_2CS	-0.112			-6.970	
		TiC	0.500000	-8.138	631
		TiS	0.500000	-5.579	1018028
Zr_2CS	-0.214			-7.806	5025
		ZrC	0.500000	-8.883	2795
		ZrS	0.500000	-6.302	7859
Hf_2CS	-0.110			-8.592	
		HfC	0.500000	-9.802	21075
		HfS	0.500000	-7.161	1206743
V_2CS	0.008			-7.167	
		V_5S_4	0.056250	-5.970	1133
		V_3S_4	0.393750	-5.531	799
		V_6C_5	0.550000	-8.475	28731
Nb_2CS	-0.003			-7.967	
		Nb_3S_4	0.437500	-6.258	12627
		Nb_6C_5	0.515625	-9.307	2760
		Nb_2C	0.046875	-9.114	569989
Ta_2CS	0.093			-8.862	
		Ta_2CS_2	0.625000	-8.007	
		Ta_2C	0.375000	-10.532	7088
Mo_2CS	0.274			-7.630	
		Mo_2C	0.375000	-9.211	1552
		MoC	0.250000	-9.236	2305
		MoS_2	0.375000	-5.708	2815
W_2CS	0.540			-8.493	
		WC	0.500000	-10.414	1894
		W	0.125000	-11.353	91
		WS_2	0.375000	-6.418	224

Table S7: Competing phases for M_2AX phases with Se as A-element.

Phase	ΔH_{cp} [eV/atom]	comp. phases	fraction	Energy [eV/atom]	MP-id
Sc_2CSe	-0.162			-6.171	
		$Sc_{15}C_{19}$	0.274194	-7.480	15957
		Sc_4C_3	0.225806	-6.938	15661
		ScSe	0.500000	-4.784	1782
Y_2CSe	-0.135			-6.405	
		Y_2C	0.125000	-6.558	1334
		Y_4C_5	0.375000	-7.589	9459
		YSe	0.500000	-5.209	2637
Ti_2CSe	-0.107			-6.588	
		TiC	0.500000	-8.138	631
		TiSe	0.500000	-4.825	571651
Zr_2CSe	-0.115			-7.468	
		ZrC	0.500000	-8.883	2795
		ZrSe	0.500000	-5.824	1183040
Hf_2CSe	-0.144			-8.228	
		$Hf_{23}Se_{25}$	0.444444	-6.273	32873
		Hf_2Se	0.055556	-7.119	1206927
		HfC	0.500000	-9.802	21075
V_2CSe	0.051			-6.794	
		V_3Se_4	0.393750	-4.784	22700
		V_5Se_4	0.056250	-5.340	1087497
		V_6C_5	0.550000	-8.475	28731
Nb_2CSe	0.041			-7.627	
		Nb_3Se_4	0.437500	-5.582	561
		Nb_6C_5	0.515625	-9.307	2760
		Nb_2C	0.046875	-9.114	569989
Ta_2CSe	0.175			-8.501	
		Ta_2CSe_2	0.625000	-7.562	
		Ta_2C	0.375000	-10.532	7088
Mo_2CSe	0.281			-7.359	
		Mo_2C	0.375000	-9.211	1552
		MoC	0.250000	-9.236	2305
		$MoSe_2$	0.375000	-5.005	1634
W_2CSe	0.549			-8.195	
		WC	0.500000	-10.414	1894
		W	0.125000	-11.353	91
		WSe_2	0.375000	-5.648	1821

Table S8: Competing phases for M_2AX phases with Te as A-element.

Phase	ΔH_{cp} [eV/atom]	comp. phases	fraction	Energy [eV/atom]	MP-id
Sc_2TeC	-0.061			-5.687	
		Sc_4C_3	0.225806	-6.938	15661
		ScTe	0.500000	-4.018	10026
		$Sc_{15}C_{19}$	0.274194	-7.480	15957
Y_2TeC	-0.093			-5.965	
		Y_4C_5	0.375000	-7.589	9459
		YTe	0.500000	-4.414	1708
		Y_2C	0.125000	-6.558	1334
Ti_2TeC	0.016			-6.082	
		Ti_3Te_4	0.218750	-3.794	15669
		Ti_5Te_4	0.281250	-4.265	7498
		TiC	0.500000	-8.138	631
Zr_2TeC	-0.048			-7.003	
		ZrTe	0.500000	-5.028	1539
		ZrC	0.500000	-8.883	2795
Hf_2TeC	-0.037			-7.728	
		Hf_5Te_4	0.375000	-5.936	12884
		$HfTe_2$	0.125000	-4.519	32887
		HfC	0.500000	-9.802	21075
V_2TeC	0.175			-6.335	
		VTe_2	0.195000	-3.501	11687
		V_9Te_8	0.255000	-4.569	1216446
		V_6C_5	0.550000	-8.475	28731
Nb_2TeC	0.129			-7.197	
		Nb_3Te_4	0.437500	-4.799	7564
		Nb_6C_5	0.515625	-9.307	2760
		Nb_2C	0.046875	-9.114	569989
Ta_2TeC	0.257			-8.051	
		Ta_2Te_2C	0.625000	-6.974	
		Ta_2C	0.375000	-10.532	7088
$TeMo_2C$	0.305			-7.022	
		Mo_2C	0.375000	-9.211	1552
		MoC	0.250000	-9.236	2305
		Te_2Mo	0.375000	-4.169	602
TeW_2C	0.536			-7.875	
		WC	0.500000	-10.414	1894
		Te_2W	0.375000	-4.759	22693
		W	0.125000	-11.353	91

References

[S1] Beckmann, O.; Boller, H.; Nowotny, H. Die Kristallstrukturen von Ta_2S_2C und Ti_4S_5 ($Ti_{0.81}S$). *Monatshefte für Chemie* **1970**, *101*, 945–955.

[S2] Boller, H.; Hiebl, K. Quaternary pseudo-intercalation phases T_x [Nb_2S_2C] (T=V, Cr, Mn, Fe, Co, Ni, Cu) and metastable Nb_2S_2C formed by topochemical synthesis. *Journal of Alloys and Compounds* **1992**, *183*, 438–443.

[S3] Majed, A.; Kothakonda, M.; Wang, F.; Tseng, E. N.; Prenger, K.; Zhang, X.; Persson, P. O. Å.; Wei, J.; Sun, J.; Naguib, M. Transition Metal Carbo-Chalcogenide "TMCC:" A New Family of 2D Materials. *Advanced Materials* **2022**, *34*, 2200574.

[S4] Sakamaki, K.; Wada, H.; Nozaki, H.; Ōnuki, Y.; Kawai, M. Topochemical formation of van der Waals type niobium carbosulfide 1T-Nb_2S_2C. *Journal of Alloys and Compounds* **2002**, *339*, 283–292.

[S5] Ohmer, D.; Qiang, G.; Opahle, I.; Singh, H. K.; Zhang, H. High-throughput design of 211-M_2AX compounds. *Physical Review Materials* **2019**, *3*, 053803.

[S6] Beckmann, O.; Boller, H.; Nowotny, H. Neue H-Phasen. *Monatshefte für Chemie* **1968**, *99*, 1580–1583.

[S7] Yang, J.; Wang, A.; Zhang, S.; Wu, H.; Chen, L. Stability and electronic properties of sulfur terminated two-dimensional early transition metal carbides and nitrides (MXene). *Computational Materials Science* **2018**, *153*, 303–308.

[S8] Björk, J.; Rosen, J. Functionalizing MXenes by Tailoring Surface Terminations in Different Chemical Environments. *Chemistry of Materials* **2021**, *33*, 9108–9118.

[S9] Zhang, L.; Tang, C.; Zhang, C.; Du, A. First-principles screening of novel ferroelectric MXene phases with a large piezoelectric response and unusual auxeticity. *Nanoscale* **2020**, *12*, 21291–21298.

[S10] Hu, C.; Yu, X.; Li, Y.; Cheng, J.; Xiao, B. M_2CS_2 (M = Sc, Y) with brand-new MXene phase: The promising candidate as the N/O-containing gases sensor and/or capturer. *Applied Surface Science* **2023**, *607*, 155104.

[S11] Nie, X.; Ji, Y.; Ding, Y. M.; Li, Y. Layer-stacking of chalcogenide-terminated MXenes Ti_2CT_2(T = O, S, Se, Te) and their applications in metal-ion batteries. *Nanotechnology* **2022**, *34*, 105704.

[S12] Tang, C.; Wang, X.; Zhang, S. Research on metallic chalcogen-functionalized monolayer-puckered V_2CX_2 (X = S, Se, and Te) as promising Li-ion battery anode materials. *Materials Chemistry Frontiers* **2021**, *5*, 4672–4681.

[S13] Zhu, J.; Chroneos, A.; Eppinger, J.; Schwingenschlögl, U. S-functionalized MXenes as electrode materials for Li-ion batteries. *Applied Materials Today* **2016**, *5*, 19–24.

[S14] Wang, S.-Y.; Pan, C.; Tang, H.; Wu, H.-Y.; Shi, G.-Y.; Cao, K.; Jiang, H.; Su, Y.-H.; Zhang, C.; Ho, K.-M.; Wang, C.-Z. Straintronic Effect on Phonon-Mediated Superconductivity of Nb 2 CT 2 (T = O, S, Se, or Te) MXenes. *The Journal of Physical Chemistry C* **2022**, *126*, 3727–3735.

[S15] Wang, Y.; Ma, Y.; Zhang, Q.; Huang, R.; Gao, B.; Li, Z.; Li, G.; Liang, F. First-principles investigation of V_2CSe_2 MXene as a potential anode material for non-lithium metal ion batteries. *Current Applied Physics* **2022**, *41*, 7–13.

[S16] Jasani, J.; Mishra, P.; Sonvane, Y. A First-principles investigation of the structural and electronic properties of Two-dimensional Hf_2CSe_2. *Materials Today: Proceedings*

[S17] Pang, X.; Wu, T.; Gu, Y.; Wang, D.; Che, X.; Sun, D.; Huang, F. Nb_2Se_2C: a new compound as a combination of transition metal dichalcogenide and MXene for oxygen evolution reaction. *Chemical Communications* **2020**, *56*, 9036–9039.

[S18] Boller, H. The Influence of Reaction Paths on Powder Metallurgical Reactions, Illustrated at the Preparation of Some Sulphide Carbides. *International Journal of Refractory Metals and Hard Materials* **1993–1994**, *12*, 195–197.

[S19] Kamysbayev, V.; Filatov, A. S.; Hu, H.; Rui, X.; Lagunas, F.; Wang, D.; Klie, R. F.; Talapin, D. V. Covalent surface modifications and superconductivity of two-dimensional metal carbide MXenes. *Science* **2020**, *369*, 979–983.

[S20] Xu, G.; Wang, J.; Zhang, X.; Yang, Z. First principles study on geometric and electronic properties of two-dimensional Nb_2CT_x MXenes. *Chinese Physics B* **2022**, 037304.

[S21] Jain, A.; Ong, S. P.; Hautier, G.; Chen, W.; Richards, W. D.; Dacek, S.; Cholia, S.; Gunter, D.; Skinner, D.; Ceder, G.; Persson, K. A. Commentary: The Materials Project: A materials genome approach to accelerating materials innovation. *APL Materials* **2013**, *1*, 011002.
